# Degradation of de‐esterified pctin/homogalacturonan by the polygalacturonase GhNSP is necessary for pollen exine formation and male fertility in cotton

**DOI:** 10.1111/pbi.13785

**Published:** 2022-02-18

**Authors:** Yuanlong Wu, Xiao Li, Yanlong Li, Huanhuan Ma, Huabin Chi, Yizan Ma, Jing Yang, Sai Xie, Rui Zhang, Linying Liu, Xiaojun Su, Rongjie Lv, Aamir Hamid Khan, Jie Kong, Xiaoping Guo, Keith Lindsey, Ling Min, Xianlong Zhang

**Affiliations:** ^1^ 47895 National Key Laboratory of Crop Genetic Improvement Huazhong Agricultural University Wuhan Hubei China; ^2^ 47895 College of Plant Science and Technology Huazhong Agricultural University Wuhan Hubei China; ^3^ 74608 Institute of Economic Crops Xinjiang Academy of Agricultural Sciences Xinjiang China; ^4^ Department of Biosciences Durham University Durham UK

**Keywords:** cotton, male sterility, exine formation, no spine pollen, polygalacturonase, de‐esterified homogalacturonan

## Abstract

The pollen wall exine provides a protective layer for the male gametophyte and is largely composed of sporopollenin, which comprises fatty acid derivatives and phenolics. However, the biochemical nature of the external exine is poorly understood. Here, we show that the male sterile line 1355A of cotton mutated in *NO SPINE POLLEN* (*GhNSP*) leads to defective exine formation. The *GhNSP* locus was identified through map‐based cloning and confirmed by genetic analysis (co‐segregation test and allele prediction using the CRISPR/Cas9 system). *In situ* hybridization showed that *GhNSP* is highly expressed in tapetum. *GhNSP* encodes a polygalacturonase protein homologous to AtQRT3, which suggests a function for polygalacturonase in pollen exine formation. These results indicate that *GhNSP* is functionally different from *AtQRT3*, the latter has the function of microspore separation. Biochemical analysis showed that the percentage of de‐esterified pectin was significantly increased in the 1355A anthers at developmental stage 8. Furthermore, immunofluorescence studies using antibodies to the de‐esterified and esterified homogalacturonan (JIM5 and JIM7) showed that the *Ghnsp* mutant exhibits abundant of de‐esterified homogalacturonan in the tapetum and exine, coupled with defective exine formation. The characterization of *GhNSP* provides new understanding of the role of polygalacturonase and de‐esterified homogalacturonan in pollen exine formation.

## Introduction

In flowering plants, the pollen wall protects the gametophyte against pathogen attack, dehydration and UV irradiation, protects the haploid male sperm cells and provides a genetic barrier via pollen recognition and adhesion to the stigma (Ariizumi and Toriyama, 2011; Shi *et al*., 2015; Xu *et al*., 2017). The highly durable pollen wall comprises two layers, an inner intine and an outer exine. The acquisition of exine is a vital adaptation for their successful land colonization by plants (Ariizumi and Toriyama, 2011; Chen *et al*., 2011). The intine is structurally simpler and is composed of cellulose, hemicellulose, pectin polymers, hydrolytic enzymes and hydrophobic proteins derived from the microspores themselves (Ariizumi and Toriyama, 2011; Huang *et al*., 2009). The exine encompasses two layers, the outer sexine (consisting of the tectum and bacula) and the inner nexine (consisting of foot layer and endexine), and is likely largely constructed of sporopollenin, which is composed of fatty acid derivatives and phenolics derived from the tapetum and is highly resistant to physical, chemical and biological degradation (Ariizumi and Toriyama, 2011; Scott *et al*., 2004; Shi *et al*., 2015; Xu *et al*., 2017). Studies also showed that the nexine may contain arabinogalactan proteins (AGPs) (Jia *et al*., 2015; Lou *et al*., 2014). Great efforts have been made to identify the biochemical nature of the exine; however, given its unusual insolubility and extreme stability, its biochemical nature is poorly understood (Shi *et al*., 2015). Recent morphological and molecular genetic studies have increased our understanding of exine development (Kim *et al*., 2010; Xu *et al*., 2017; Yang *et al*., 2014). As one of the important features for species identification, the sexine determines the surface morphology of pollen and is usually sculpted in a taxon‐specific manner into, for example, spines and other features of pollen ornamentation. In angiosperms, the basic structure and the formation of the exine appears to be strongly conserved. However, the fine structure of the exine and the relative thickness of each stratum vary between species (Gong *et al*., 2015).

Pollen wall development can be divided into three stages: primexine formation (Chang *et al*., 2012), the transition of primexine to exine (Ariizumi and Toriyama, 2011) and mature pollen wall formation (de Azevedo Souza *et al*., 2009; Dobritsa *et al*., 2009, 2010; Kim *et al*., 2010). As a hallmark of the beginning of pollen wall development, primexine formation begins at the tetrad stage. After the microspores are released from the callose wall, the primexine is rapidly transformed into the exine. However, whether it is degraded or becomes part of the exine is unknown. The nexine can be further divided into two layers. However, relatively little is known about the molecular mechanisms of nexine formation. Studying nexine formation not only provides insight into the molecular mechanisms of pollen development but also provides benefits for the utilization of heterosis in crops. When the remnants of the tapetum are deposited as tryphine to fill the exine cavities, the mature pollen wall is formed.

Here, we describe a male sterile mutant in cotton (*Ghnsp*) that is associated with defective formation of the nexine and the spines. The gene was identified using map‐based cloning and confirmed by the co‐segregation tests and allele prediction using the CRISPR/Cas9 system. *GhNSP* encodes a polygalacturonase protein and is highly expressed in the tapetal layer at stage 8 (the microspores released from the tetrad). Further biochemical and immunofluorescence studies indicate that de‐esterified homogalacturonan accumulates abundantly in tapetal cells and microspores of *Ghnsp* mutant plants. These results suggest that GhNSP degrades de‐esterified pectin/homogalacturonan, and that this degradation plays a pivotal role in exine formation and male fertility in cotton.

## Results

### Map‐based cloning of the fertility restorer *GhNSP* in the 1355A male sterile line

**Figure 1:**
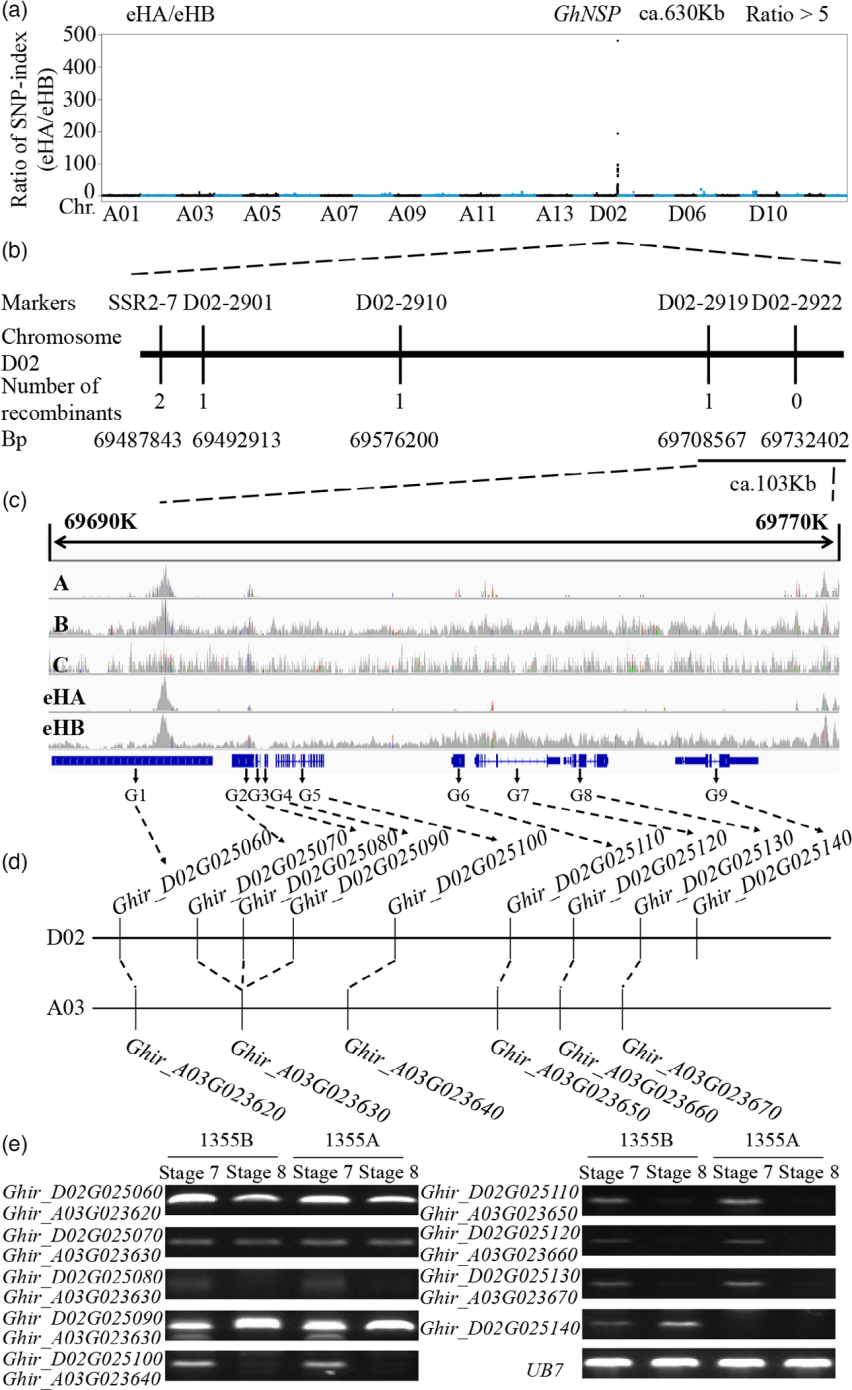
Map‐based cloning of *GhNSP*. (a) Mapping‐by‐sequencing of the loci underlying *GhNSP* in a 1355A × E22 F_2_ population. Pooled DNA from male sterile plants (eHA) and male fertile plants (eHB) was sequenced, and the ratios of the SNP‐index (eHA/eHB) along chromosomes are shown. The regions about 630 kb at the end of chromosome D02 represents the peak of ratios. (b) Fine mapping of the *GhNSP* region using a NIL line, with 1840 plants, shows that the simple sequence repeat (SSR) marker D02‐2922 is co‐segregated with the *GhNSP* locus. The thick bar represents the genomic region; numbers underneath the bars indicate the numbers of recombinants between *GhNSP* and the molecular marker. (c) Integrative genomics viewer shot of sequencing reads coverage in 1355A, 1355B, 1355C and two pools DNA from the 1355A × E22 F_2_ population and indicated that a deletion region about 62 kb existed on the candidate region in the male sterile plants. A, 1355A; B, 1355B; C, 1355C. (d) Synteny analysis of the nine candidate genes in chromosome D02and A03 of *G. hirsutum* (There are large area translocation between chromosomes D02 and A03 in *G. hirsutum* genomes, which leads to synteny area within the *GhNSP* region in the chromosome D02 and A03). Among these nine genes, only *Ghir_D02G025140* does not have homologous gene pairs in the A03. (e) Using RT‐PCR, the expression pattern analysis of the nine candidate genes and *ubiquitin7* (*UB7*) were performed in the anthers of 1355AB lines during the stage 7 (tetrad stage) and stage 8 (the microspores released from the tetrad) and indicated that the expression of *Ghir_D02G025140* significantly decreased in 1355A line as compared to the other candidate genes.

Our previous study showed that the 1355A male sterile line displayed complete male sterility with no spine pollen (*nsp*) but normal vegetative development (Wu *et al*., 2015). To isolate the fertility restorer gene *GhNSP* for the 1355A male sterile line, 1355A male sterile plants were pollinated by wild‐type pollen (Emian 22), all of the F_1_ progeny were fertile, and the F_2_ population displayed 3 : 1 segregation of fertile to sterile plants (1105 : 331, χ^2^ = 2.81, *P* < 0.05), indicating that sterility is controlled by a single recessive gene (Figure S1A). Using 1355A × Emian 22 F_2_ populations, the pools of male sterile plants (eHA) and male fertile plants (eHB) were subjected to next‐generation sequencing (NGS)‐based bulked segregation analysis (BSA), and 515 040 078 and 489 159 624 paired‐end 150‐bp clean reads for pools of male sterile plant (32 plants) and male fertile plant (32 plants) were obtained respectively. A candidate region of about 630 kb at the end of chromosome D02 was identified (Figure 1a).

To fine map the *GhNSP* locus, simple sequence repeat (SSR) markers were developed at the end of chromosome D02 by referencing the genome sequence of *G. hirsutum* cv TM‐1 (Wang *et al*., 2015; Zhang *et al*., 2015), and five polymorphic SSRs were identified with the segregating population derived from the near‐isogenic line (NIL) 1355AB developed by Wu *et al*. through many generations of backcrossing (Wu *et al*., 2015; Figure S1B). By using a population of 1840 plants from the two‐type lines 1355AB (935:905, χ^2^ = 0.4571, *P* < 0.05) and the five polymorphic SSRs, referring to the genome sequence, the *GhNSP* locus was narrowed to ca.103 kb flanked by D02‐2919 (Figure 1b). By resequencing analysis of 1355A (*Ghnsp*/*Ghnsp*), 1355B (*GhNSP*/*Ghnsp*), 1355C (*GhNSP*/*GhNSP*), the male sterile plants pool (eHA) and male fertile plants pool (eHB), it was found that a deletion region of about 62 kb existed in the candidate region in the male sterile plant 1355A and male sterile pool eHA (Figure 1c). Based on high‐quality reference genome sequence of tetraploid cotton TM‐1 (Wang *et al*., 2019), synteny analysis of the candidate region was performed, and syntenic blocks were identified in the deletion region between chromosomes D02 and A03 in the deletion region (Figure S2A). There is large area translocation between chromosomes D02 and A03 in *G. hirsutum* genomes as described by Wang *et al*. (2019). Nine genes were found in the candidate region, among which only *Ghir_D02G025140* does not have homologous gene pairs in the A03 (Figure 1D). To confirm this, gene‐specific primers for *Ghir_D02G025130*, *Ghir_D02G025140* and *Ghir_A11G011460* (*ub7*) and DNA derived from 1355A, 1355B, YZ1, Jin668, TM‐1 and *G. barbadense* cv Xinhai‐35 (Figure S2B) were used for the molecular test and found that *Ghir_D02G025140* was deleted in 1355A plants, without homologous gene pairs.

The expression of the nine candidate genes was checked between male fertile line and male sterile line using conserved primers, and it was found that the expression of *Ghir_D02G025140* was significantly reduced compared to that of the other candidate genes (Figure 1e), which suggested that *Ghir_D02G025140* is a candidate for *GhNSP*.

### Genetic analysis of the fertility restorer *GhNSP*

**Figure 2:**
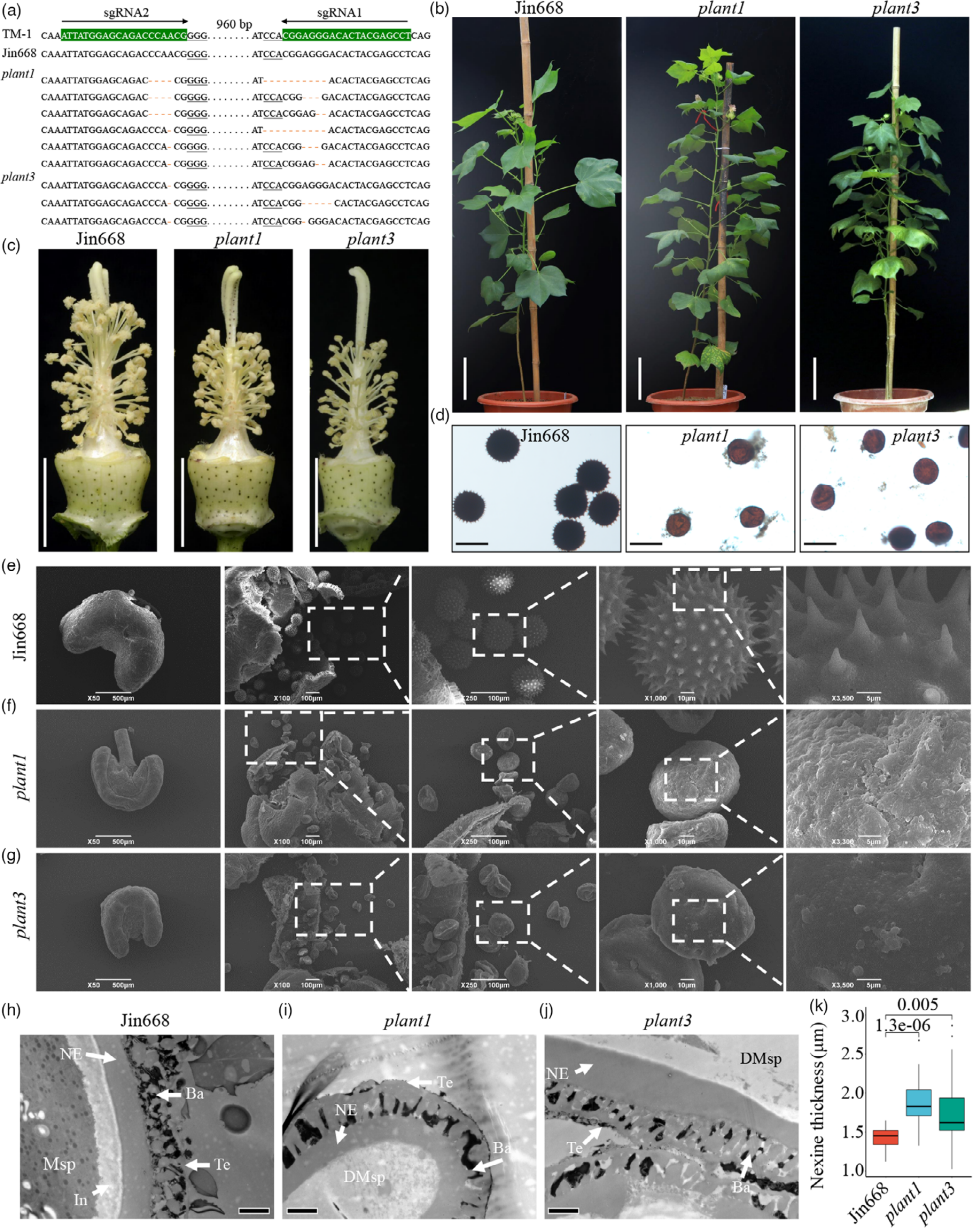
Genotypic and phenotypic comparison between the Jin668 (WT) and the mutations created by the CRISPR/Cas9 system. (a) Genome editing in the mutant of *plant1* and *plant3* is shown. The sgRNA target sites and the PAM regions are highlighted in green background and underlined respectively. PAM, protospacer adjacent motif. (b) The WT and mutant plants are shown at full‐bloom stage. (c) The flowers of WT and mutant plants are shown, with petals removed. The anthers of mutants are indehiscence. (d) The pollen grains of WT and mutant plants stained with 1% I_2_‐KI solution. The black pollen grains are fertile, and the red pollen grains are sterile. (e–g) Scanning electron microscopy analysis of pollen grains of the Jin668 (e), the mutant *plant1* (f) and *plant3* (g). Compared with those of Jin668 pollen grains, the mutant pollen grains lacked spines in the pollen wall. (h–j) Transmission electron microscopy analysis of pollen walls from the Jin668 (h) and the mutant *plant1* (i) and *plant3* (j). (k) The nexine wall thickness of pollen walls from the Jin668 and the mutants *plant1* and *plant3* at stage 12. At least 10 pollen grains of each genotype were analyzed. The *P*‐value was calculated by using the Student *t*‐test (*n* > 10). The error bars represent standard deviations (SDs). Msp, microspores; DMsp, degenerated microspores; In, intine; NE, nexine; Ba, bacula; Te, tectum. Bars, 10 cm in (b) and (c); 100 μm in (d); 600 μm, 100 μm, 100 μm, 10 μm, 5 μm in (e–g); 2 μm in (h–j).

To confirm the prediction, we designed a CRISPR/Cas9 construct with two sgRNAs targeting the regions in the second and third exons of the *Ghir_D02G025140* gene (gene length, 8428 bp; coding sequence length, 1563 bp; including three exons and two introns) to create mutants (Figure S3A), and four transgenic plants were obtained (Figure S3B). Using the Hi‐TOM platform (Liu *et al*., 2019) to track mutations created by CRISPR/Cas9, we found that all four transgenic plants contained mutations (Figure S3C,D). *plant4* developed abnormally (Figure S3E), so the mutants *plant1* and *plant3* were used for further study (Figure 2a). Morphological comparison of the mutants (*plant1* and *plant3*) with wild type (WT, Jin668) revealed that vegetative and floral development appeared normal in both (Figure 2b). However, stamen filaments were shorter and anthers failed to dehisce in the two mutant plants at flowering (Figure 2c).

I_2_‐KI staining showed that mutant pollen grains lacked spines and failed to stain indicative of complete male sterility (Figure 2d). Scanning electron microscopic analysis showed that at stage 12, the WT anthers and pollen were normal, and the exine with spines was formed (Figure 2e). However, in the mutant plants, the pollen was shrunken and the exine lacked spines (Figure 2f–g). Transmission electron microscopic analyses also showed that the sexine and nexine were normal in the WT plant (Figure 2h); while in the mutant plants, the sexine was smooth (without spines), the nexine was significantly thickened and the pollen wall was lacking intine at stage 12 (Figure 2i–k), phenotypically similar to male sterile plant 1355A (Wu *et al*., 2015). *plant1* and *plant3* were hybridized with WT, and in the F_1_ generation, three plants contained the CRISPR/Cas9 transgene derived from *plant1* and four from *plant3*; and two and three F_1_ plants were CRISPR/Cas9‐free from *plant1* and *plant3* respectively (p1p2‐p1p4 and p3p1‐p3p4 representing the CRISPR/Cas9‐positive individuals derived from *plant1* and *plant3* mutant lines respectively; p1n1/p1n3 and p3n1‐p3n3 representing the CRISPR/Cas9‐free individuals derived from *plant1* and *plant3* mutant lines respectively; Figure S4A–C). Phenotypic analysis showed that all the CRISPR/Cas9‐containing plants were male sterile, and all the CRISPR/Cas9‐free plants were male fertile (Figure S5). Genotypic analysis confirmed that all the CRISPR/Cas9‐containing plants contained mutations at the *Ghir_D02G025140* gene locus, and all the CRISPR/Cas9‐free plants were heterozygous at the *Ghir_D02G025140* gene locus (Figure S6), indicating that the *Ghir_D02G025140* gene co‐segregates with male sterility.

**Figure 3:**
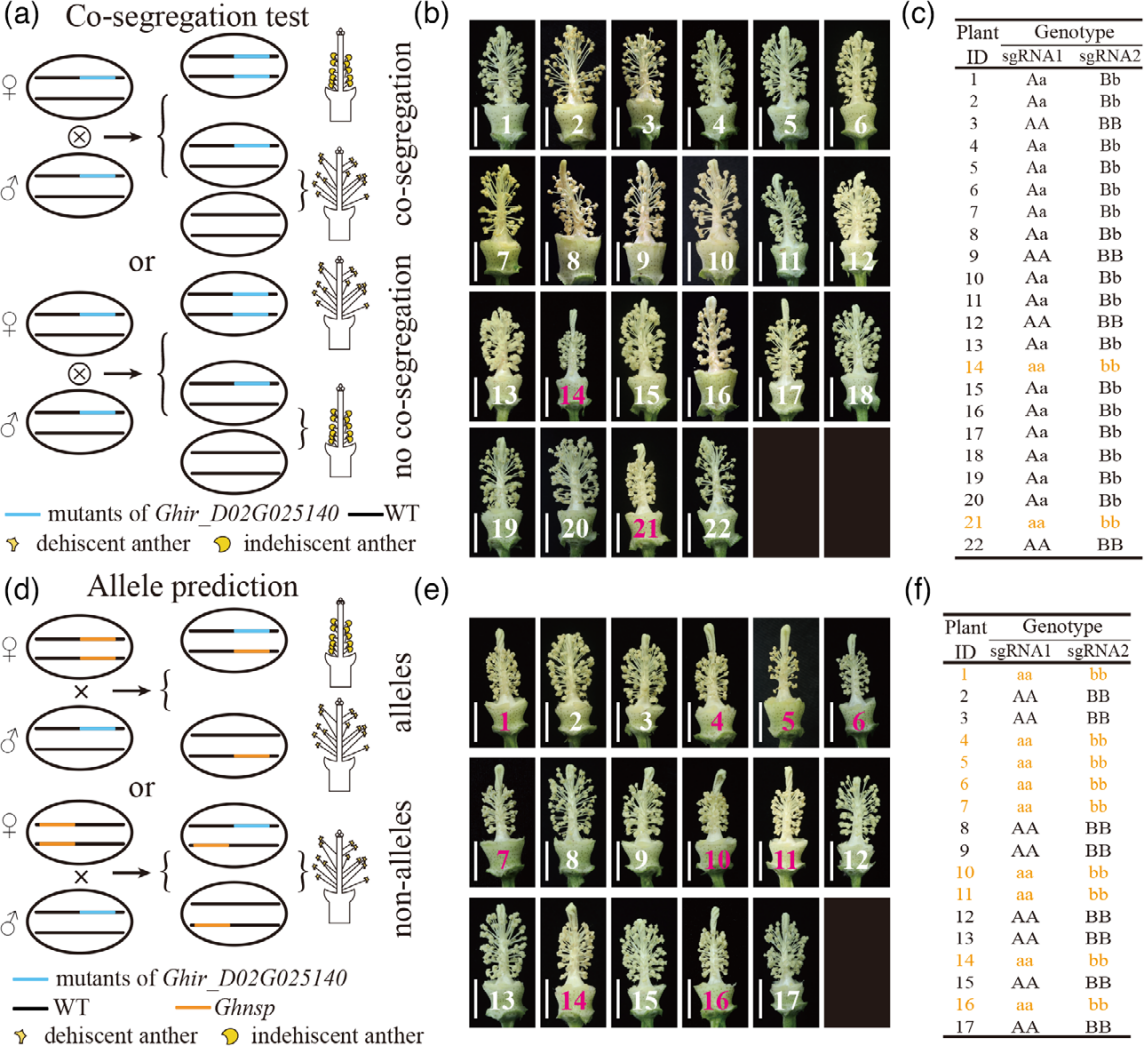
Co‐segregation test and allele prediction of the fertility restorer *GhNSP*. (a) A schematic for the co‐segregation test. The male fertile anther is dehiscent, and the male sterile anthers is indehiscent. (b) The flowers of the individuals of S_1_ population derived from p1n1 plant, with petals removed. The number with white and bright red represent male fertile plant and male sterile plant respectively. (c) The Hi‐TOM sequence results of the individuals of S_1_ population derived from p1n1 plant are summarized. p1n1, the CRISPR/Cas9‐free plants derived from the F_1_ generation of *plant1* mutant (T_0_) crossed with WT. A\a represents sgRNA1 site; B\b represents sgRNA2 site; AA and BB stand for wild type in *GhNSP* locus; Aa and Bb stand for heterozygous genotype in *GhNSP* locus; aa and bb stand for homozygous mutant genotype in *GhNSP* locus. The orange characters represent the genotype of male sterile plants. (d) A schematic for allele prediction. The male fertile anther is dehiscent, and the male sterile anthers is indehiscent. (e) The flowers of the individuals of C_1_ population derived from p1n1 plant, with petals removed. The number with white and bright red represent male fertile plant and male sterile plant, respectively. (f) The Hi‐TOM sequence results of the individuals of C_1_ population derived from p1n1 plant are summarized in the table. p1n1, the CRISPR/Cas9‐free plants derived from the F_1_ generation of *plant1* mutant (T_0_) crossed with WT. A\a represents sgRNA1 site; B\b represents sgRNA2 site; AA and BB stand for wild type in *GhNSP* locus; Aa and Bb stand for heterozygous genotype in *GhNSP* locus; aa and bb stand for homozygous mutant genotype in *GhNSP* locus. The orange characters represent the genotype of male sterile plants.

To further confirm this result, p1n1 and p3n3 plants (CRISPR/Cas9 free and heterozygous at the *Ghir_D02G025140* locus derived from *plant1* and *plant3* respectively) were selected. Progeny were generated through self‐pollination to produce segregation populations for the *Ghir_D02G025140* locus (S_1_ populations; Figure 3a). Phenotypic analysis of individuals of the S_1_ populations showed that two of 22 individuals derived from p1n1 (Figure 3b) and four of 22 individuals from p3n3 (Figures S7 and S8) were male sterile (with anther indehiscence and inactive pollen) in the S_1_ populations. Genotypic analysis of individuals of the S_1_ populations revealed that the *Ghir_D02G025140* gene co‐segregates with male sterility (Figure 3c, Table 1 and Figure S9).

**Table 1:** The results of the co‐segregation test and allele prediction

Test	Plant	Phenotypic (plant no.)		χ^2^	*P* value	Genotype (plant no.)			χ^2^	*P* value
		Male fertile	Male sterile			*GhNSP/GhNSP*	*GhNSP/Ghnsp*	*Ghnsp/Ghnsp*		
Co‐segregation	p1n1 selfed	20	2	2.18 (3:1)	>0.10	4	16	2	4.91 (1:2:1)	>0.05
	p3n3 selfed	18	4	0.24 (3:1)	>0.75	3	15	4	2.80 (1:2:1)	>0.1
Allele prediction	p1n1 crossed with 1355A	8	9	0 (1:1)	>0.90	0	8	9	0 (1:1)	>0.90
	p3n3 crossed with 1355A	9	10	0 (1:1)	>0.90	0	9	10	0 (1:1)	>0.90

These results reveal that the *Ghir_D02G025140* gene is a fertility restorer gene in cotton; however, it was not known whether *Ghir_D02G025140* is also the fertility restorer gene of the 1355A male sterile line. To confirm this, the populations of the allele predictive test were generated by using the selected plants (p1n1 and p3n3) to cross with 1355A plants (C_1_ populations), and then, the male sterile phenotypes and genotypes were investigated in the individuals of C_1_ populations (Figure 3d). It was found that nine of 17 individuals from p1n1 (Figure 3e) and nine of 19 individuals from p3n3 (Figure S10) were male sterile in the C_1_ populations, confirmed by the pollen grains staining with I_2_‐KI solution (Figure S11). Genotypic analysis of individuals of the C_1_ populations revealed that *Ghir_D02G025140* is allelic with the 1355A fertility restorer gene *GhNSP* (Figure 3f, Table 1 and Figure S12).

To assess the genetic background of the co‐segregation test and allele prediction populations (S_1_ and C_1_ populations), we observed leaf shape and found that the *Ghnsp* mutant plants and the plants of S_1_ populations showed normal leaves, the 1355A plants had okra leaves and the plants of C_1_ populations had subokra leaves (Figure S13A). Molecular test using the SSR marker SWU07345, which is the marker co‐segregating with the okra leaf trait, revealed the correctness of the genetic background of the population used for the co‐segregation test and allele prediction (Figure S13B). Therefore, the genetic analysis proved that *Ghir_D02G025140* is *GhNSP*.

### *GhNSP* is an anther‐specific gene mainly expressed in the tapetum

**Figure 4:**
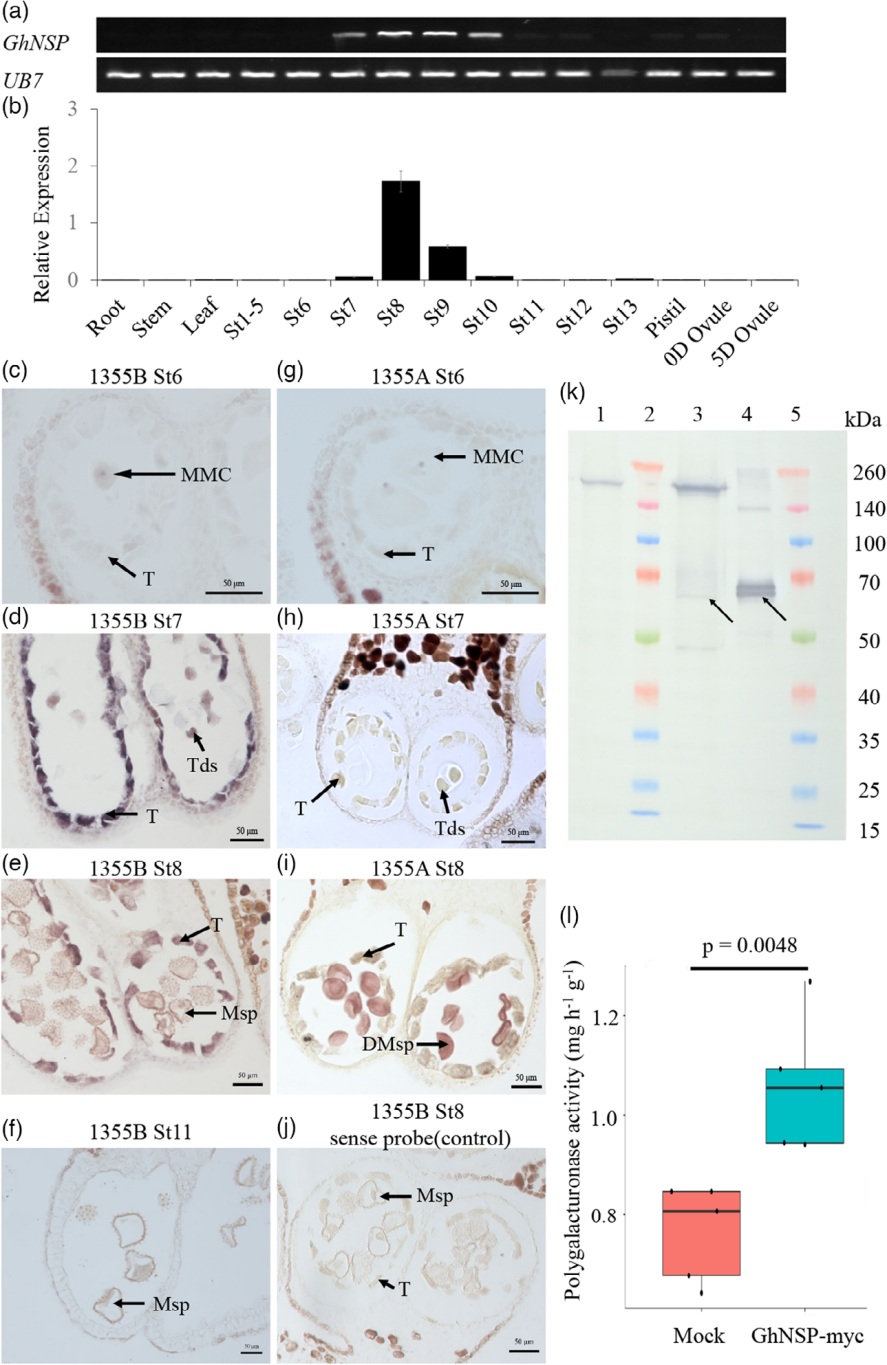
Expression profiles and protein function of *GhNSP*. (a and b) Spatial and temporal expression analysis of *GhNSP* by RT‐PCR (A) and qRT‐PCR (b). *ubiquitin7* (*UB7*) served as a control. (c–j) *In situ* hybridization of *GhNSP* in anthers of two‐type lines 1355AB. Locules from the anther section of the 1355B plants (c–f and j) and 1355A plants (g–i) at stage 6 (c and g), stage 7 (d and h), stage 8 (e, i and j) and stage 11 (f). The anthers from 1355A plants served as negative controls. (h) No signal was detected at the microspore stage (stage 8) with Gh*NSP* sense probe from the anther section of the 1355B plants. (k) Extracts from leaves infiltrated with *Agrobacterium* carrying the *GhNSP‐myc* construct were used to perform the expression‐level analysis of GhNSP‐myc protein by Western blotting with antibody against the myc tag. The extracts containing total proteins were purified using the Pierce Magnetic c‐myc‐Tag IP/Co‐IP Kit, and the anti‐myc and non‐anti‐myc proteins were collected and named as purified and unbound proteins respectively. The unbound proteins served as a negative control. Lane 1, unbound proteins; Lane 2 and Lane 5, Marker (kDa); Lane 3, extracts containing total proteins; Lane 4, purified proteins. The arrows indicate the GhNSP‐myc proteins. (l) The polygalacturonase activity was detected. Mock: The total proteins extracted from *N. benthamiana* leaves were infiltrated with *Agrobacterium* without recombinant plasmid. GhNSP‐myc: The total proteins extracted from *N. benthamiana* leaves were infiltrated with *Agrobacterium* containing recombinant plasmid GhNSP‐myc (myc‐tagged at the C‐terminus of GhNSP). GhNSP‐myc recombinant proteins showed stronger polygalacturonase activity than the proteins from Mock. Data are depicted in the source data, and the *P*‐value was calculated by using the Student *t*‐test (*n* = 5). St, anther stage; T, tapetal layer; MMC, microspore mother cells; Tds, tetrads; Msp, microspores; DMsp, degenerated microspores. Bars, 50 μm in (c, e–g and i–j); 20 μm in (d and h).

RT‐PCR and quantitative real‐time PCR (qPCR) revealed that *GhNSP* is expressed in the early anther developmental stage, starting at stage 7, peaking at stage 8 and dissipating by stage 11 but is not detectable in the root, stem, leaf, pistil, 0‐day ovule, 5‐day ovule or the other anther developmental stages (Figure 4a,b). To more precisely detect the spatial and temporal patterns of *GhNSP* expression, RNA *in situ* hybridization on floral sections of 1355B male fertile line was performed. At early stage 6, no *GhNSP* expression was detectable (Figure 4c). At stage 7 and stage 8, *GhNSP* RNA was detected in the tapetum of 1355B (Figure 4d,e). At stage 11, *GhNSP* RNA could not be detected (Figure 4f). RNA *in situ* hybridization using the floral sections of the 1355A male sterile line was also performed at stages 6, 7 and 8, which served as negative controls (Figure 4g–i). The sense probe was used to detect the background levels of signal in the 1355B at stage 8 (Figure 4j). These results demonstrated that *GhNSP* is expressed in the tapetal cells, consistent with an essential role in anther development.

### *GhNSP* encodes a polygalacturonase protein

To gain further information about Ghir_D02G025140, we used the full‐length Ghir_D02G025140 protein sequence (520 aa) to search for orthologous sequences in the Phytozome v12 public database (https://phytozome.jgi.doe.gov/pz/portal.html) with BLASTP. A maximum likelihood phylogenetic tree analysis grouped the sequences into two clades (Figure S14). Thecc1EG027717, GSVIVG01029528001, Sobic.006G212400, Zm00008a006302, LOC_Os04g52320, Sphfalx0012s0113, Pp3c1_12100 and Pp3c9_20390 were grouped in the first clade, which was further divided into three subclades. The three subclades are mosses, monocotyledons and dicotyledons. Interestingly, the other clade was all dicotyledonous plants. This analysis indicated that GhNSP may represent a conserved and divergent polygalacturonase member in dicotyledonous plant species.

InterProScan search showed that GhNSP contains a consensus pectate_lyase_3 domain, homologous to the QUARTET 3 (QRT3) polygalacturonase of *Arabidopsis* (Figure S15). To determine whether GhNSP exhibits polygalacturonase activity, the plasmid expressing GhNSP tagged with the myc tag at its C‐terminus (*GhNSP‐myc*) was infiltrated into *N. benthamiana* leaves via *Agrobacterium tumefaciens* strain *GV3101*. The part of extracts from leaves then infiltrated with *Agrobacterium* carrying the *GhNSP‐myc* construct was used to perform the expression‐level analysis of GhNSP‐myc protein by Western blotting (Figure 4k). When the GhNSP‐myc protein had been expressed, the left extracts were measured the polygalacturonase activity and compared it with the negative control (mock, extracts from leaves infiltrated with *Agrobacterium* without a construct). The assay measured the ability to hydrolyze polygalacturonic acid, creating galacturonic acid whose reducing aldehyde group reacts with the reagent DNS (3,5‐dinitrosalicylic acid) to form a reddish‐brown product (Guan *et al*., 2020). The absorbance value was higher in the assay with GhNSP fusion protein compared with the control, demonstrating that GhNSP exhibits polygalacturonase activity (Figure 4l).

### Disabled GhNSP caused the accumulation of de‐esterified homogalacturonan in early‐stage anthers

**Figure 5:**
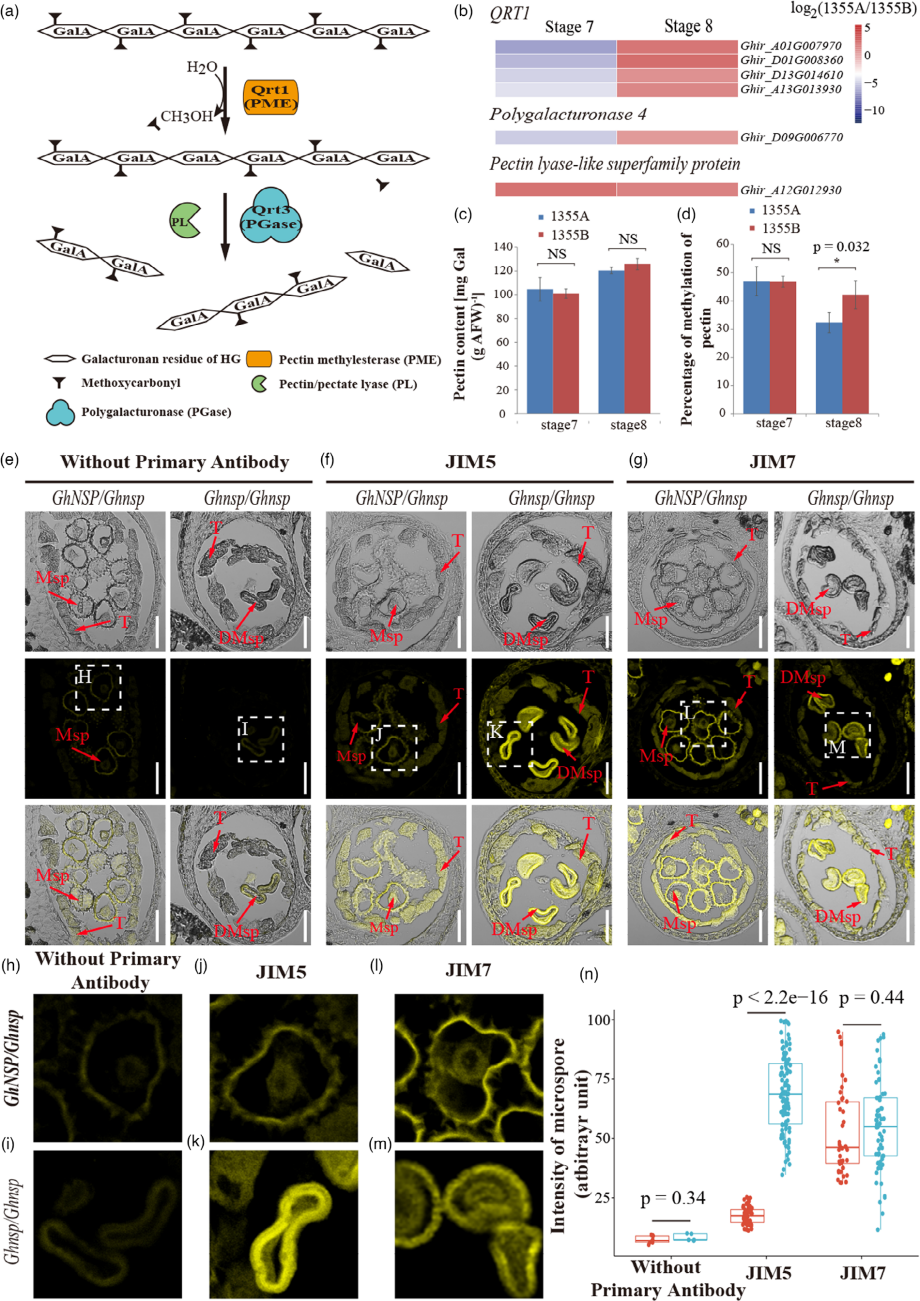
GhNSP functions in the de‐esterified homogalacturonan degradation. (a) A model for homogalacturonan degradation in pollen development described by Francis *et al*. (2006) with minor modifications. The de‐esterified pectin was generated by pectin demethylesterification by PMEs and then cleaved by the PG. PMEs, pectin methylesterase; PG, polygalacturonase. (b) Heatmap of pectin metabolism pathway‐related genes in the anther of 1355AB lines at stage 7 and stage 8 was shown. (c) Polygalacturonic acid content in the anther of 1355AB lines at stage 7 and stage 8. The *P*‐value was calculated by using the Student *t*‐test (n = 3), NS, not significant. (d) The degrees of methylation of pectin in the anther of 1355AB lines at stage 7 and stage 8. The *P*‐value was calculated by using the Student *t*‐test (n = 3). NS, not significant; *, *P* < 0.05. (e–g) Immunofluorescence studies of pectin associated with microspores of the male fertile (*GhNSP*/*Ghnsp*) and the male sterile (*Ghnsp*/*Ghnsp*) plants. Sections were stained without primary antibody (e), with antibodies against de‐esterified pectin (f) and esterified pectin (g). T, tapetal layer; Msp, microspores; DMsp, degenerated microspores. Bars, 60 μm in (e–g). (h–m) Enlarged regions of microspores in (e–g) were showed. (N) Immunofluorescence signals intensity of microspore derived from the male fertile (*GhNSP*/*Ghnsp*) and the male sterile (*Ghnsp*/*Ghnsp*) plants. The *P*‐value was calculated by using the Student *t*‐test (*n* > 4 in sample without primary antibody, n > 30 in sample with JIM5 and JIM7).

Previous studies showed that homogalacturonan is the main component of pectins, and polygalacturonase (PG, QRT3) functions in the pectin metabolism pathway (Rhee *et al*., 2003). Pectin is demethylesterified by pectin methylesterase (PME) to produce de‐esterified pectin/homogalacturonan and then cleaved by PG or pectate lyase (PL) to form galacturonic acid (Figure 5a). To confirm the transcriptional changes of related genes in the anther of male sterile line 1355A and male fertile line 1355B, we searched our previous RNA‐Seq raw data (Wu *et al*., 2015), and results showed that the expression of four *QRT1* (*PME*) genes and the *POLYGALACTURONASE‐4* gene was down‐regulated at stage 7, and up‐regulated at stage 8; however, the pectin lyase‐like superfamily protein was up‐regulated at stages 7 and 8 (Figure 5b). We speculated that de‐esterified homogalacturonan might accumulate abundantly in *Ghnsp* mutant. To investigate this, the polygalacturonic acid content and the degree of pectin methylation were measured in 1355B and 1355A anthers at stages 7 and 8. No significant differences were found in pectin contents between 1355B and 1355A anthers during the stages analyzed (Figure 5c). However, the percentage of methylation of pectin was significantly reduced in the 1355A anthers at stage 8 (Figure 5d), suggesting de‐esterified homogalacturonan accumulates in the early‐stage anthers of *Ghnsp* mutant.

### High presence of de‐esterified homogalacturonan accumulate in the tapetum and pollen exine of the *Ghnsp* mutant

**Figure 6:**
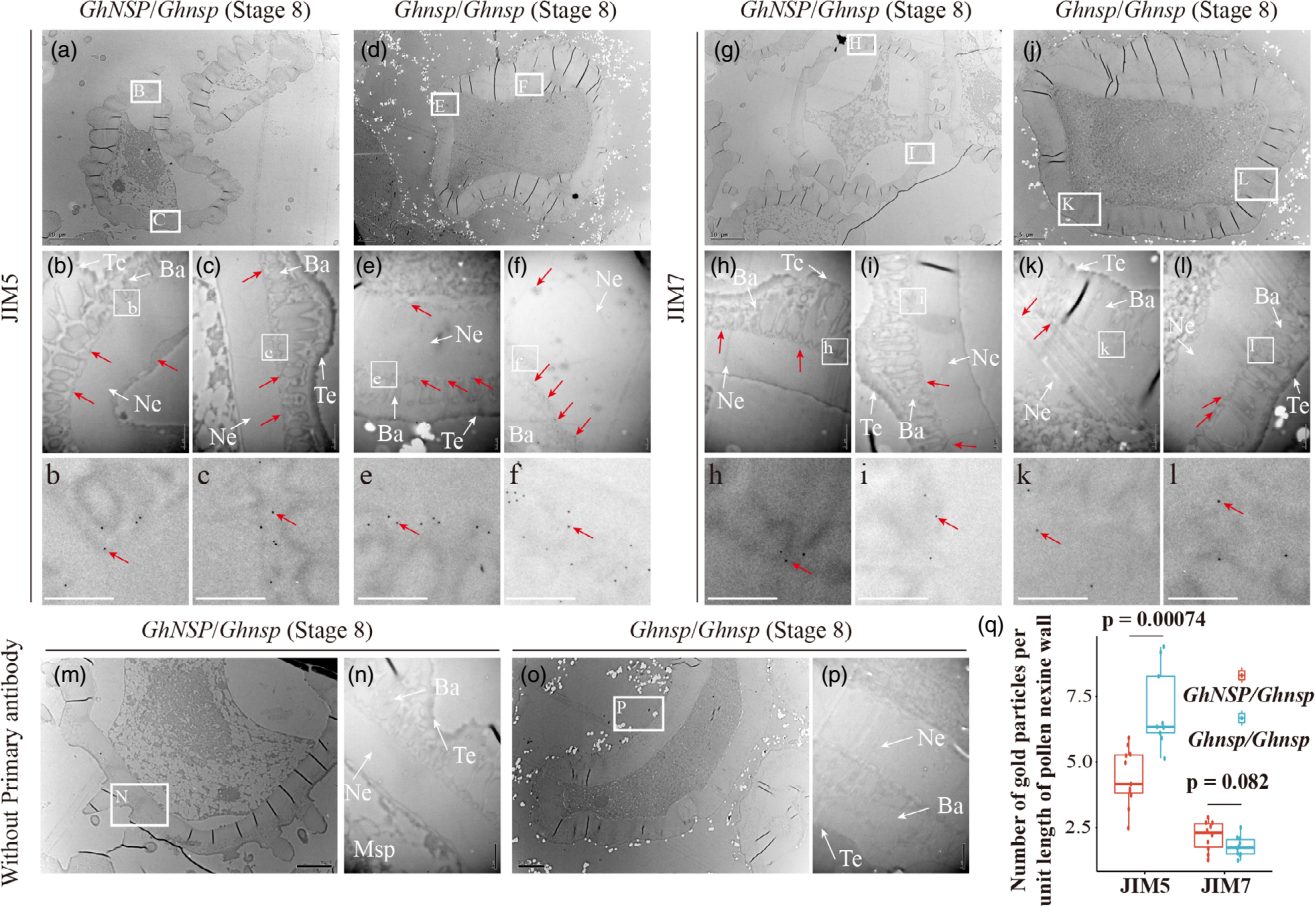
Localization of de‐esterified pectin and esterified pectin in pollen wall by using immune electron microscopy. (a–p) Immuno electron microscopy to detect the pectin associated with microspores of the male fertile (*GhNSP*/*Ghnsp*, a, b, e–g and k–m) and the male sterile (*Ghnsp*/*Ghnsp*, c, d, h–j and n–p) plants at stage 8. Ultrathin sections were incubated with antibodies against de‐esterified pectin (a–f), esterified pectin (g–l) and without primary antibody (m–p), (b) and (c), enlarged regions outlined in (a). Figure (E) and (f), enlarged regions outlined in (d). Figure (h) and (i), enlarged regions outlined in (g). Figure (k) and (l), enlarged regions outlined in (j). Figure (n), enlarged regions outlined in (m). Figure (p), enlarged regions outlined in (o). Figure (b, c, e, f, h, i, k and l), enlarged regions outlined in b, c, e, f, h, i, k and l respectively. Figure (q) Quantification of gold particles following immuno TEM. By using ImageJ, the number of gold particles per square micron in pollen nexine wall multiplied by the thickness of pollen nexine, which is the number of gold particles per unit length of pollen nexine wall, was calculated. The *P*‐value was calculated by using the Student *t*‐test (*n* > 5). Red arrows point to representative gold particles. More gold particle‐labelled anti‐JIM5 antibody was found in the pollen wall of male sterile plants, compared with male fertile plants. Msp, microspores; DMsp, degenerated microspores; NE, nexine; Ba, bacula; Te, tectum. Bars, 10 μm in (a and g), 5 μm in (d, j, m and o), 1 μm in (b, c, h, i, k, l, n and p) and 0.5 μm in (e, f, b, c, e, f, h, i, k and l).

To explore the location of de‐esterified homogalacturonan in the early‐stage anthers of *Ghnsp*, antibodies that react with de‐esterified homogalacturonan (JIM5) and esterified homogalacturonan (JIM7) were used to detect homogalacturonan associated with developing pollen at stage 8 by immunofluorescence studies of anther tissue sections of *Ghnsp*/*Ghnsp* (homozygous mutant) and *GhNSP*/*Ghnsp* (heterozygous) plants. Although weak spontaneous fluorescence was detected in microspores and tapetal cells of *GhNSP*/*Ghnsp* plants without antibodies (Figure 5e), the fluorescence was strong with the JIM5 antibody against de‐esterified homogalacturonan in tapetal cells and microspores of mutant plants, compared with *GhNSP*/*Ghnsp* plants (Figure 5f). Compared with *GhNSP*/*Ghnsp* plants, no significant differences in fluorescence using JIM7 antibody against esterified homogalacturonan was detected in mutant plants (Figure 5g). These results were further confirmed by quantitative results of microspore fluorescence by using ImageJ (Figure 5h–n). We speculated that GhNSP can degrade the de‐esterified homogalacturonan in tapetal cells and anther locule, which might provide proper de‐esterified homogalacturonan content for the formation of the normal pollen wall, with the *Ghnsp* mutation leading to a high presence of de‐esterified homogalacturonan and to the formation of defective pollen wall. To confirm this, immuno transmission electron microscopy was performed. Gold particle‐labelled anti‐JIM5 antibody was strongly and primarily located in the pollen exine of mutant plants at stage 8 (Figure 6a–p), similar to the results of the immunofluorescence study (Figure 5e–g). Furthermore, the number of gold particles per square micrometre in the pollen nexine wall multiplied by the thickness of pollen nexine was calculated by using ImageJ, and showed that gold particle‐labelled anti‐JIM5 antibody bounds strongly in the pollen exine of mutant plants (Figure 6q). These results demonstrated that GhNSP exhibits polygalacturonase activity, and the high presence of de‐esterified homogalacturonan accumulate in the pollen exine of the *Ghnsp* mutant.

## Discussion

### GhNSP plays an important role in pollen nexine and spine development

In flowering plants, pollen development involves a series of characteristic changes in the cell walls, and the pollen wall is most remarkable and unique (Owen and Makaroff, 1995). The intine and the exine are the two principal layers of the pollen wall (Blackmore *et al*., 2007), and the exine is divided into sexine and nexine. Recent investigations revealed that defective pollen walls lead to male sterility in 1355A plants, as shown by failing to form spines on the pollen wall surface, a thicker nexine and lack of intine (Wu *et al*., 2015). In this work, *GhNSP,* encoding a polygalacturonase protein, is identified as the restorer gene for male sterility in 1355A plants, and its function was confirmed by CRISPR/Cas9 system‐mediated mutant analysis. Polygalacturonase activity is important for proper exine patterning in *Arabidopsis*, in addition to degradation of the primary cell wall (Dobritsa *et al*., 2011; Rhee *et al*., 2003). In this study, a thicker nexine is seen in the *Ghnsp* mutant, and spines fail to form on the pollen wall surface (Figure 2). These results indicated that the polygalacturonase activity of GhNSP plays an important role in nexine and spine formation during pollen development. *GhNSP* is mainly expressed in the tapetum (Figure 4). However, the intine is secreted by microspores (gametophytic origin) (Huang *et al*., 2009; Schnurr *et al*., 2006). The cytoplasm is degraded in the pollen grains of 1355A plants (Wu *et al*., 2015), and previous studies indicated that the nexine acts as a place for the accumulation of substances during intine formation (Lou *et al*., 2014). Thus, *GhNSP* seems to be an indirect factor of the intine formation.

### De‐esterified homogalacturonan and the degradation products of de‐esterified pectin/homogalacturonan might be essential components of the exine layer formation in the pollen wall

Pectin is the most structurally complex family of polysaccharides and has a range of functions such as in cell wall porosity, cell adhesion, pollen tube elongation, plant defence and others (Yang *et al*., 2013). Polygalacturonase (PG), which is one of the pectin‐degrading enzymes, has been isolated from anthers of tobacco (*Nicotiana tabacum*), *Brassica napus*, Populus and *Arabidopsis* (Crouch, 1990; Rhee *et al*., 2003; Tebbutt *et al*., 1994; Yang *et al*., 2013; Zhang *et al*., 2012) and is considered to be involved in cell wall synthesis and regulation. In addition, PG in a pollen tube may dissolve cell walls in the style, allowing pollen tubes to pass through (Huang *et al*., 2009). PG can also act on a pollen tube’s own cell wall, thus accelerating its growth (Huang *et al*., 2009).

**Figure 7:**
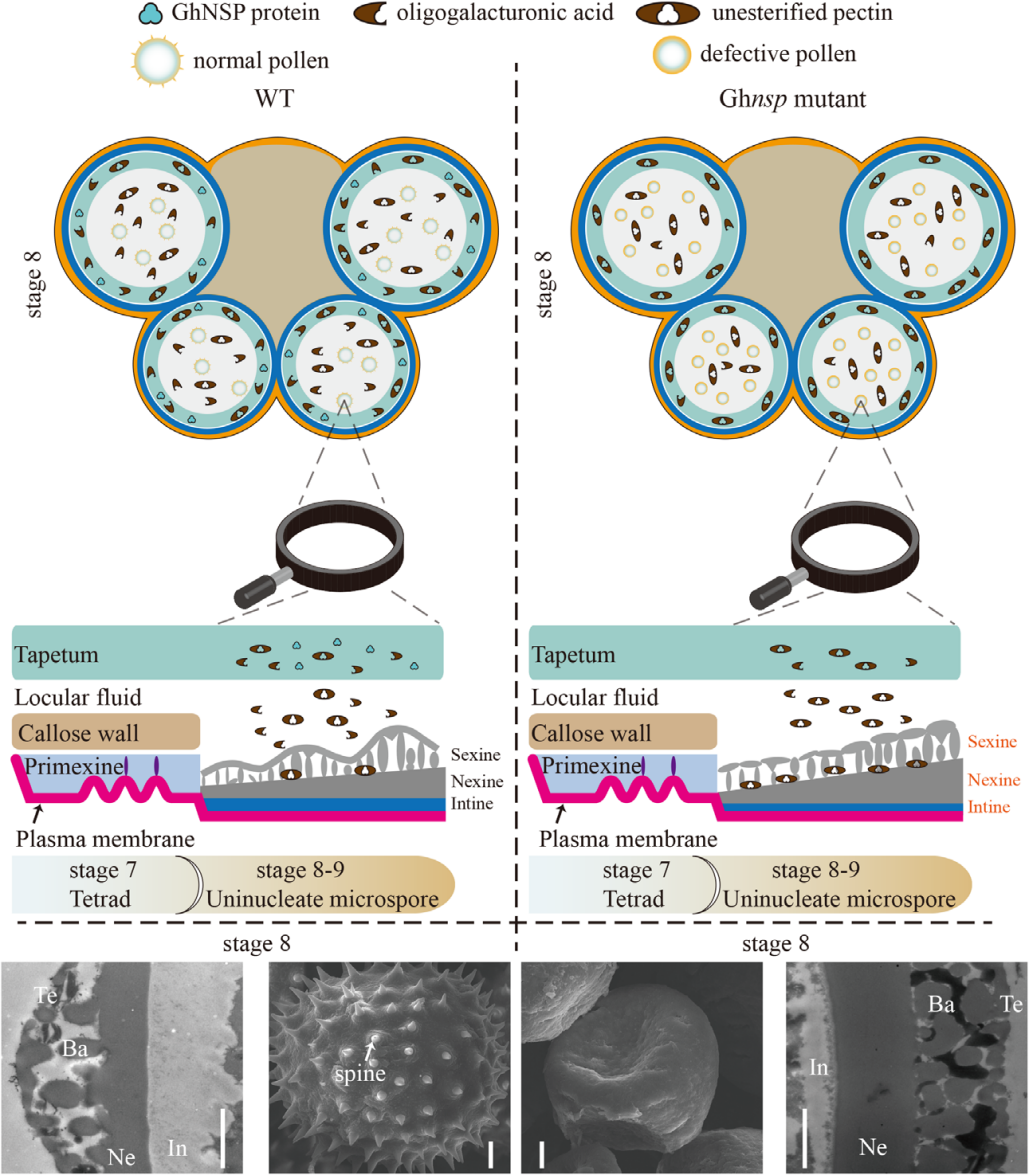
Schematic model illustrating spine and nexine formation in the anther of WT and *Ghnsp* mutant. The legend at top shows the GhNSP protein, oligogalacturonic acid, de‐esterified pectin, normal pollen and defective pollen. Scanning electron microscope (SEM) and transmission electron microscope (TEM) results of the pollen of male fertile and male sterile plants at stage 8 are shown at the bottom. Defective pollen is characterized by failure to form spines on the pollen wall surface, with a thicker nexine at stage 8. *Ghnsp* mutant caused abundantly accumulation of de‐esterified homogalacturonan in the tapetum and exine. The orange in the pollen indicates exine, and the peacock blue in the pollen indicates intine. Yellow ring, epidermis cells; aqua blue ring, endothecium cells; light blue ring, tapetal cells.

In this study, *GhNSP*, which is the restorer gene for male sterility in 1355A plants, was successfully cloned in cotton using a map‐based cloning strategy. *GhNSP* encodes a member of the polygalacturonase protein family protein and is homologous to *AtQRT3* of *Arabidopsis*. It is reported to function in degrading the pollen mother cell wall during microspore development in *Arabidopsis* (Rhee *et al*., 2003). The antibody against de‐esterified pectin stained the primexine during the early microspore stage of pollen development in both the wild type and the *qrt3‐1* mutant in Arabidopsis (Rhee *et al*., 2003), and recent study showed that the AtQRT3 delayed degradation of the tetrad pectin wall (Shi *et al*., 2021). Moreover, exine phenotypes of *qrt‐dex1* and *qrt‐dex2* indicated that the polygalacturonase activity of AtQRT3 may be important for proper exine patterning (Dobritsa *et al*., 2011). Unfortunately, no further studies have been performed to study the molecular mechanism of AtQRT3 function in proper exine patterning. Our results showed GhNSP exhibits polygalacturonase activity and participates in exine formation. In addition, the de‐esterified pectin/homogalacturonan accumulates by pectin demethylesterification involving PMEs (for example, QRT1) and then is cleaved by the PG (for example, QRT3) in *Arabidopsis* (Francis *et al*., 2006). Biochemical analysis showed a reduction in methylated pectin in the 1355A anthers at stage 8 in the absence of changes in total pectin content, which would be consistent with mutant anthers accumulating de‐esterified pectin. Immunofluorescence studies of pectin/homogalacturonan composition during stage 8 of anther development stage in the *Ghnsp* mutant demonstrated that more de‐esterified homogalacturonan components persist around the microspores and tapetal cells, which supports the view that GhNSP exhibits polygalacturonase activity. Combined with the results of genetic analysis, we demonstrate that *GhNSP*, encoding a polygalacturonase protein, plays a pivotal role in nexine and spine formation during pollen development in cotton. We propose a model in which the *Ghnsp* mutant abundantly accumulates de‐esterified homogalacturonan in tapetum and pollen exine, which coupled with the thicker nexine, fails to form spines on the pollen wall surface and lacks intine, so causing male sterility (Figure 7).

However, the substrates and products of GhNSP during pollen wall development have not been clearly defined, and more sophisticated assays will be necessary to fully characterize the processes. Interestingly, although the *Arabidopsis* QRT3 and GhNSP both have polygalacturonase activity, they showed different phenotypes in the pollen wall. *Arabidopsis A*t*QRT3* is specifically and transiently expressed in the tapetum during the tetrad stage (Rhee *et al*., 2003), while the expression of *GhNSP* appeared during the tetrad stage and increased along the developmental stages, peaked at stage 8, and then, declined, with longer expression than that of *AtQRT3*. Based on these results, we speculated that the different stage of *QRT3* gene expression may lead to the different phenotype. Systematic study of their differences will help to explore the mechanism of pollen wall formation.

In addition, nexine layer formation involves AGPs (AGP6, AGP11, AGP23, AGP24 and AGP40), and it is proposed that glycoproteins might be essential components of the nexine layer in the pollen wall (Jia *et al*., 2015). Thus, AGPs may act as plasticizers by decreasing pectin cross‐linking (Lamport and Kieliszewski, 2005). Interestingly, among the five *AGPs* mentioned above, only *AGP23* has a homologous gene in *G. hirsutum*, which was down‐regulated at stage 7, and slightly up‐regulated at stage 8 in 1355A male sterile plants (Figure S16). This suggests that GhNSP may indirectly affect the expression of *AGP23*, leading to defective pollen wall formation and causing male sterility in 1355A male sterile plants. Nexine formation takes place after sexine formation, and the *Ghnsp* mutant shows defective spine formation of the sexine (Ariizumi and Toriyama, 2011), and thus, the degradation products of de‐esterified pectin/homogalacturonan by GhNSP may be involved in spine formation. When *GhNSP* is mutated, the degradation of de‐esterified pectin/homogalacturonan is blocked, the de‐esterified pectin/homogalacturonan accumulates in tapetum and microspores, and defective exine lacking spines and with thicker nexine is produced. Based on those results, we propose that the degradation products of de‐esterified pectin/homogalacturonan by GhNSP might serve as the components of the spines in the cotton pollen wall and that the de‐esterified homogalacturonan in tapetal cells and around the developing pollen could be a component of nexine.

### CRISPR/Cas9 system provides a strategy for breeding various male sterile lines in *G. hirsutum and G. barbadense*

A male sterility system for hybrid rice breeding and seed production was constructed, which overcame the intrinsic problems of both CMS and PTGMS systems (Chang *et al*., 2016). However, it is difficult to use this system in cotton, for it needs strong restorer genes for male sterility, which are lacking in cotton. In this study, we isolated *GhNSP*, which makes it possible to construct this male sterility system in *G. hirsutum*. To investigate whether the *Ghnsp* mutant via the CRISPR/Cas9 system can be transferred into other cotton species to breed male sterile lines, the mutant *plant3* (as maternal parent; T_0_) was crossed with *G. barbadense* cv xinhai‐35. Progeny was analyzed for their associated phenotypes and the genotypes of *GhNSP* locus. There are both male sterile and male fertile plants in the F_1_ progeny (Figure S17A). There are some variations between *Ghir_D02G025140* and the *Gbar_D02G025630/Gbar_D04G020900* gene pairs, but the sgRNA sequences were the same, indicating the CRISPR/Cas9 system works in *G. barbadense*. The Hi‐Tom results showed that *Gbar_D02G025630* and *Gbar_D04G020900* gene pairs co‐segregate with male sterility (Figure S17B,C). This work provides a potential sterile/restorer system for heterosis utilization by male sterility both in *G. hirsutum* and *G. barbadense*. These results also indicated that the *GhNSP*‐CRISPR/Cas9 system maintains functionality when transferred from *G. hirsutum* into a *G. barbadense* background, and the function of the *GhNSP* was conserved between species.

## Experimental procedures

### Plant material and growth conditions

Cotton plants were grown in the field during the normal cotton planting season and in the greenhouse during the winter in Wuhan, China. The F_2_ mapping population was generated from a cross between 1355A (male sterile line) and Emian 22 (fertile cultivar) for BSA cloning. The development of the NILs of upland cotton used for fine mapping has been described previously (Wu *et al*., 2015). The p1n1 and p3n3 plants (CRISPR/Cas9 free and heterozygous at the *Ghir_D02G025140* locus derived from *plant1* and *plant3* respectively) were generated from backcross between *Ghnsp* mutant (T_0_) and the WT (Jin668). The populations for co‐segregation tests (S_1_ populations, the first progeny of p1n1 or p3n3 plant through self‐pollination) and allele prediction (C_1_ populations, the first progeny of p1n1 or p3n3 plant crossed with 1355A plant) were generated (Figure S18). All transgenic plant and their generations were grown in the greenhouse at 28–35 °C/20–28 °C day/night in Wuhan. All samples collected from plants were immediately deep‐frozen in liquid and stored at −70 °C.

### Mapping‐by‐sequencing

To map the causal mutations in the 1355A male sterile line, we generated second‐generation (F_2_) populations by crossing the 1355A male sterile line with the cultivar Emian 22. From a total of 1436 [male sterile line 1355A × Emian 22] F_2_ plants, 32 male sterile plants and 32 male fertile plants were selected as the two BSA samples, and the DNA extraction was performed for each plant using the Plant Genome Extraction Kit (TIANGEN Biotech, Beijing, China). An equal amount of DNA from male sterile plants and male fertile plants was each pooled for NGS. The DNA from the leaves of 1355A (*Ghnsp/Ghnsp*), 1355B (*GhNSP/Ghnsp*) and 1355C (*GhNSP/GhNSP*) were also sequenced. The sequencing of two pools and 1355A, 1355B and 1355C was performed by Beijing Genomics Institute (Shenzhen, China). For Emian 22, the raw data of resequencing were downloaded from the NCBI Sequence Read Archive (SRA) under accession number SRP080913 (Wang *et al*., 2017). The clean reads mapped to the reference genome of *G. hirsutum* (Wang *et al*., 2019) using BWA software (version 0.7.10‐r789). SNPs were called with Samtools and GATK programs, and the method for bulk segregant analysis was the same as that described previously (Soyk *et al*., 2017). The male sterile/male fertile SNP ratios were calculated in 100‐kb windows sliding 10 kb and plotted across the 26 cotton chromosomes using R (https://www.r‐project.org/).

### CRISPR/Cas9‐mediated mutation

The CRISPR/Cas9 system was used to create mutant alleles of *GhNSP* (*Ghir_D02G025140.1*, http://www.cottonfgd.org/profiles/transcript/Ghir_D02G025140.1/) in Jin668, an easy transformation cultivar (Li *et al*., 2018). The pRGEB32‐GhU6.7‐NPT II‐*GhNSP* plasmid was constructed as described previously (Wang *et al*., 2018). In brief, two single‐guide sgRNAs (sgRNA1 in 4460–4483 bp; sgRNA2 in 3476–3498 bp) were designed using the CRISPR‐P tool (http://crispr.hzau.edu.cn/CRISPR2/) (Liu *et al*., 2017). The fragments containing tRNA‐sgRNA1 (AGGCTCGTAGTGTCCCTCCGTGG in the third exon of *GhNSP*) fusion and gRNA‐tRNA‐sgRNA2 (ATTATGGAGCAGACCCAACGGGG in the second exon of *GhNSP*) fusion were obtained using pGTR as template via the primer *GhNSP*‐CRISPR (Table S1) and cloned into the pRGEB32‐GhU6.7‐NPT II vector. The construct was introduced into *Agrobacterium tumefaciens* strain *EHA105* and was used to transform cotton Jin668, as described previously (Li *et al*., 2018). Transgenic plants regenerated were first examined for the presence of the transgene using primer set u6 and inf *GhNSP*‐CRISPR, and the transgenic plants were then subjected to the Hi‐TOM platform (Liu *et al*., 2019) using primer set *GhNSP*‐Hi‐TOM to evaluate whether mutation occurred. The mutant lines (termed *plant1* and *plant3*) were back‐crossed with WT Jin668 to generate the F1, and the CRISPR/Cas9‐free F_1_ generations were used to generate S_1_ populations through self‐pollination and to generate an allele prediction population (C_1_ populations) through crossing with 1355A plants. The genotype of *GhNSP* in the S_1_ and C_1_ populations was then analyzed using the Hi‐TOM platform. The primers used are listed in Table S1.

### Microscopy and histology

Plants and floral organs were photographed with a Canon EOS 80D digital camera. To analyze pollen fertility during anthesis, pollen grains were stained with I_2_‐KI solution (1‐g I_2_ and 3‐g KI dissolved in 100‐mL distilled water) before photography using a Zeiss Axio Scope‐A1 microscope.

For scanning and transmission electron microscopy, anthers were collected from the male sterile plants (*Ghnsp*/*Ghnsp*) and male fertile plants (*GhNSP*/*Ghnsp*) simultaneously and immediately prefixed in 2.5% glutaraldehyde (v/v)/0.1‐M phosphate buffer (pH 7.2) at 4 °C and then vacuum infiltrated until the samples sank to the bottom of container as described by Wu *et al*. (2015). One part of fixed anthers was dehydrated in an ethanol series, transferred to isoamyl acetate, dried to the critical point and observed using a JSM‐6390/LV scanning electron microscope. The other part of fixed anthers was washed, postfixed, dehydrated, then infiltrated with Spurr resin (SPI, SPI Chem, West Chester, PA). After polymerization at 65 °C for 48 h, the samples were cut into ultrathin sections (60‐ to 70‐nm thick), and then, stained and observed using a Hitachi transmission electron microscope (H‐7650; Hitachi, Japan) at 80 kv. The thickness of nexine wall was measured using Nano measurer 1.2 software (http://emuch.net/html/201402/7022970.html). The measurements were performed for more than 12 points at random for each sample. At least 10 pollen grains of each genotype were analyzed.

### *In situ* hybridization

A 478‐bp fragment of *GhNSP* cDNA was amplified from the cDNA, which was made from the stage 8 anther of 1355B plants, with the primers *GhNSP*‐S and *GhNSP*‐AS (Table S1). The PCR product was cloned into the pGEM‐T‐Easy vector (Promega, Madison, WI, USA) and sequenced. Sense and antisense probes were transcribed in vitro from the T7 or SP6 promoter with respective RNA polymerases using the digoxigenin RNA‐labelling kit (Roche). Tissue sections were prepared as described by Min *et al*. (2013). In brief, samples were fixed in FAA [10% formalin, 5% acetic acid and 50% ethanol (v/v) in RNAase‐free water]. After dehydration and embedding of the tissue in paraffin wax, the sample blocks were sectioned into 10‐μm slices using the microm HM 340E microtome (Thermo Scientific, Waltham, MA, USA) and were applied to RNAase‐free glass slides. The sections were then dewaxed, rehydrated, prehybridized, hybridized and visualized as described by manufacturer’s instructions. Hybridization was detected by using the antidigoxigenin‐alkaline phosphatase conjugate (Roche, Mannheim, Germany), visualized by incubation with NBT/BCIP stock solution (Roche), and images were captured using a Zeiss Axio Scope‐A1 microscope.

### Expression of GhNSP in *N. benthamiana*

Full‐length cDNA of *GhNSP* without a stop codon in the pDONR™/Zeo vector was cloned into vector pGWB417 by LR recombination reaction to produce *GhNSP‐myc* (myc‐tagged at the C‐terminus of GhNSP) recombinant plasmid. The recombinant plasmid was then transformed into the *A. tumefaciens* strain *GV3101*, which was then infiltrated into *N. benthamiana* leaves. The infiltrated plants were grown at 25 °C for 60 h, and then, the infiltrated leaves were collected and instantly frozen in liquid nitrogen. About 2 g of infiltrated leaf tissues was ground into powder in liquid nitrogen and homogenized in 2 mL of extraction buffer. The soluble fusion protein was purified using the Pierce Magnetic c‐myc‐Tag IP/Co‐IP Kit (Thermo Scientific, Waltham, MA, USA) following the manufacturer’s instructions and was detected using Western blotting.

### Enzyme assays

Polygalacturonase hydrolyzes pectic acid to form galacturonic acid, which has a reducing aldehyde group and reacts with the 3,5‐dinitrosalicylic acid (DNS) reagent to form a reddish‐brown substance. There is a characteristic peak at 540 nm, and the polygalacturonase activity can be calculated by measuring the change in absorbance at 540 nm (Guan *et al*., 2020).

The *N. benthamiana* leaves were infiltrated with *Agrobacterium* containing recombinant plasmid (*GhNSP‐myc*, as described in the ‘expression of GhNSP in *N. benthamiana*’ experimental procedures), to produce GhNSP‐myc recombinant proteins for polygalacturonase enzyme assays. *N. benthamiana* leaves infiltrated with *Agrobacterium* without recombinant plasmid were used as a control. Polygalacturonase activity of GhNSP was measured using the Polygalacturonase Assay Kit (Solarbio Life Sciences, Beijing, China) following the manufacturer’s instructions. The infiltrated *N. benthamiana* leaves were harvested for protein extraction with the extraction buffer from the Kit. The mixtures were then centrifuged (4 °C, 16 000 ***g***, 10 min), and the supernatant was collected. Supernatant (30 μL) was mixed with 120‐μL reagent A (contain polygalacturonic acid) and incubated at 40 °C water bath for 30 min, then mixed with 150 μL reagent B (contain DNS reagent), incubated for 5 min at boiling water bath and monitored at 540 nm using EnSpire ELIASA (Perkinelmer, Waltham, MA, USA). The sample with polygalacturonase enzyme inactivated by boiling was served as blank control for each sample. Enzyme activity is defined as the decomposition of pectic acid per g of the infiltrated leaves at 40 °C, pH 6.0 to produce 1 mg of galacturonic acid as an enzyme activity unit. Five biological replicate assays were performed.

### Pectin extraction

Approximately 100 mg (fresh weight) of anther tissue was ground into a fine powder in liquid nitrogen for these assays. Detailed methods were described by Peng *et al*. (2000). Cotton anther tissues were extracted with ammonium oxalate (0.5% w/v, 2.5 mL, Sigma‐Aldrich) at 100 °C for 1 h. Reaction mixtures were centrifuged for 5 min at 3000 ***g*** at room temperature. After centrifuging, the supernatant was carefully collected, and the precipitation was washed once with 2.5‐mL 0.5% ammonium oxalate. All supernatant was collected.

### Polygalacturonic acid assays

Polygalacturonic acid assays were carried out as described by Kumar *et al*. (2014) with minor modifications. The 0.2‐mL pectin extract was added to 1‐mL borate sulphuric acid reagent (0.5% sodium tetraborate in concentrated sulfuric acid) under ice‐cold conditions. The mixtures were vortexed and heated on a steam bath at 100 °C for 5 min. After cooling in an ice bath, 200‐μL supernatant was used to measure the absorbance at 520 nm using EnSpire ELIASA (Perkinelmer, Waltham, MA, USA). The other supernatant was mixed with 100 μL of 3‐phenylphenol (0.15% 3‐phenylphenol), allowed to stand for 10 min and monitored at 520 nm, employing galacturonic acid (Supelco, Bellafonte, PA, USA) as a standard. Error bars represent the standard errors. Three biological replicates were performed.

### Pectin methylation assays

Pectin methylation assays were carried out as described by Klavons and Bennett (1986) with minor modifications. Samples were added to 0.5 mL of a solution containing 10‐mm CuSO4, and 0.1 mL of 1 m NaOH was added to achieve saponification. The reaction mixture was left at 4°C for 2 h. Reaction mixtures were centrifuged for 10 min at 8000 ***g*** at room temperature. The supernatant was carefully collected. After saponification, 0.25‐mL supernatant was diluted to 0.5 mL with phosphate buffer (PH 7.5), with 0.5‐mL reagent A solution added (1 unit of alcohol oxidase from *Pichia pastoris* diluted to 1 mL with phosphate buffer, Sigma‐Aldrich, Darmstadt, Germany) and incubated at 25 °C for 25 min. One millilitre reagent B solution was then added to the mixture (0.02‐M 2,4‐pentanedione in 2.0‐M ammonium acetate and 0.05‐M acetic acid, Sinopharm Chemical Reagent Co., Ltd, Shanghai, China) and mixed well. The reaction was incubated at 60 °C for 15 min in bath. After cooling in an ice bath, 200‐μL supernatant was used to measure the absorbance at 412 nm using EnSpire ELIASA (Perkinelmer, Waltham, MA, USA). A blank sample was included as control. Error bars represent the standard errors. Three biological replicates were performed.

### Localization of de‐esterified homogalacturonan and esterified homogalacturonan

To determine the pectic polysaccharides in anthers of male sterile plants (*Ghnsp*/*Ghnsp*) and male fertile plants (*GhNSP*/*Ghnsp*), immunofluorescence labelling was performed as described by Rhee and Somerville (1998). JIM5 and JIM7 antibodies (Agrisera, Vännäs, Sweden) were used to detect de‐esterified homogalacturonan and esterified homogalacturonan respectively. The anthers of male sterile plants and male fertile plants during stage 8 were collected and immediately fixed in 4% paraformaldehyde/phosphate‐buffered saline (PBS pH 7.4, 2.7‐mm KCl, 2‐mm KH_2_PO_4_, 137.0‐mm NaCl, 10.0‐mm Na_2_HPO_4_) buffer at 4 °C. Detailed experimental processes of cross‐sections were performed as previously described (Wu *et al*., 2015). After three rinses with PBS buffer (pH 7.4), the cross‐sections were incubated with the primary monoclonal antibody JIM5 or JIM7 diluted to 1 : 10 in PBS buffer with 0.8% bovine serum albumin (BSA), overnight at 4°C. Subsequently, cross‐sections were rinsed in PBS buffer three times (5 min each) and incubated with TRITC‐conjugated secondary antibodies (Agrisera, Sweden) at 1 : 100 concentrations in PBS buffer for 2 h in darkness at room temperature. Control samples were incubated with antibody dilution buffer omitting the primary antibody (Yu and Zhao, 2012). Fluorescence signals were observed using a confocal microscope (Olympus FV1200). The fluorescence signals of microspore were quantitated by ImageJ (https://imagej.nih.gov/ij/docs/). More than four microspores were analyzed in sample without primary antibody. At least 30 microspores were analyzed in sample with JIM5 and JIM7.

To further study the localization of de‐esterified homogalacturonan by transmission electron microscope, anthers were fixed in a solution of 0.5% glutaraldehyde and 2% paraformaldehyde. The detailed procedures were performed as described by Hu *et al*. (2017). The primary monoclonal antibody was JIM5 or JIM7, and the secondary antibodies was 12‐nm colloidal gold AffiniPure donkey anti‐Mouse IgG (H + L) (EM Grade, Jackson ImmunoResearch, West Grove, PA, USA). To better quantify the content of immune gold particles, the number of gold particles per square micron in pollen nexine wall multiplied by the thickness of pollen nexine, which is the number of gold particles per unit length of pollen nexine wall, was calculated using ImageJ (https://imagej.nih.gov/ij/docs/).

## Conflicts of interest

The authors declare no conflict of interest.

## Author contributions

Y.L.W., J.K., X.P.G., L.M. and X.L.Z. conceived and designed the research. Y.L.W., X.L., J.Y., S.X., R.Z., L.Y.L., H.H.M., R.J.L and A.H.K. carried out the experiment. X.P.G. provided the 1355A/B/C lines. J.K. provided the xinhai‐35 line. Y.L.W., C.H.B., Y.Z.M. and Y.L.L. analyzed the data of sequencing. Y.L.W. and X.L. wrote the manuscript. L.M., K.L. and X.L.Z. revised the manuscript. All authors read and approved the final manuscript.

## Supporting information


**Figure S1** Populations developed for map‐based cloning of the fertility restorer *GhNSP* for 1355A male sterile line.
**Figure S2** Synteny analysis and molecular test of the candidate region.
**Figure S3** A schematic for generating the mutant of *GhNSP*.
**Figure S4** PCR and Southern blotting analysis in the individuals of the F_1_ generation of *Ghnsp* mutants crossed with WT (Jin668).
**Figure S5** Phenotypic comparison between the individual of the F_1_ generation of the transgenic plants (T_0_) crossed with Jin668.
**Figure S6** Genotypic analysis of the individual of the F_1_ generation of the transgenic plants (T_0_) crossed with Jin668.
**Figure S7** Phenotypic analysis of the individuals of S_1_ population derived from p3n3 plant.
**Figure S8** Pollen phenotypic analysis of the individuals of the S_1_ populations derived from p1n1 plant (22 individuals) and p3n3 plant (22 individuals).
**Figure S9** Genotypic analysis of the individuals of the S_1_ populations derived from p1n1 and p3n3.
**Figure S10** Phenotypic analysis of the individuals of C_1_ population derived from p3n3.
**Figure S11** Pollen phenotypic analysis of the individuals of C_1_ populations derived from p1n1 and p3n3.
**Figure S12** Genotypic analysis of the individuals of C_1_ population derived from p1n1 and p3n3.
**Figure S13** Molecular test for the population for the co‐segregation test (S_1_ populations) and allele prediction (C_1_ populations).
**Figure S14** Phylogenetic tree of GhNSP (Ghir_D02G025140, red box) and its homologs from other species.
**Figure S15** Using InterProScan to search conserved domains of the GhNSP protein.
**Figure S16** The expression pattern analysis of the AGP23 at 1355AB lines during the stage 7 and stage 8.
**Figure S17** The strategy for breeding various male sterile lines in Gossypium barbadense.
**Figure S18** Population developed for the co‐segregation test and allele prediction.
**Table S1** Oligonucleotides used in this study.
