# Supplementary material for: Degradation of de‐esterified pctin/homogalacturonan by the polygalacturonase GhNSP is necessary for pollen exine formation and male fertility in cotton
Source: Plant Biotechnol J. 2022 Feb 18;20(6):1054–68. doi: 10.1111/pbi.13785 (PMC9129075; doi:10.1111/pbi.13785)
Supplement: Supplementary file 1 — Figure S1 Populations developed for map‐based cloning of the fertility restorer GhNSP for 1355A male sterile line. Figure S2 Synteny analysis and molecular test of the candidate region. Figure S3 A schematic for generating the mutant of GhNSP. Figure S4 PCR and Southern blotting analysis in the individuals of the F1 generation of Ghnsp mutants crossed with WT (Jin668). Figure S5 Phenotypic comparison between the individual of the F1 generation of the transgenic plants (T0) crossed with Jin668. Figure S6 Genotypic analysis of the individual of the F1 generation of the transgenic plants (T0) crossed with Jin668. Figure S7 Phenotypic analysis of the individuals of S1 population derived from p3n3 plant. Figure S8 Pollen phenotypic analysis of the individuals of the S1 populations derived from p1n1 plant (22 individuals) and p3n3 plant (22 individuals). Figure S9 Genotypic analysis of the individuals of the S1 populations derived from p1n1 and p3n3. Figure S10 Phenotypic analysis of the individuals of C1 population derived from p3n3. Figure S11 Pollen phenotypic analysis of the individuals of C1 populations derived from p1n1 and p3n3. Figure S12 Genotypic analysis of the individuals of C1 population derived from p1n1 and p3n3. Figure S13 Molecular test for the population for the co‐segregation test (S1 populations) and allele prediction (C1 populations). Figure S14 Phylogenetic tree of GhNSP (Ghir_D02G025140, red box) and its homologs from other species. Figure S15 Using InterProScan to search conserved domains of the GhNSP protein. Figure S16 The expression pattern analysis of the AGP23 at 1355AB lines during the stage 7 and stage 8. Figure S17 The strategy for breeding various male sterile lines in Gossypium barbadense. Figure S18 Population developed for the co‐segregation test and allele prediction. Table S1 Oligonucleotides used in this study. [file PBI-20-1054-s001.pdf]

Supplementary Information

Degradation of de-esterified pectin/homogalacturonan by the polygalacturonase GhNSP is necessary for pollen exine formation and male fertility in cotton

Yuanlong Wu[1], Xiao Li[2], Yanlong Li[1], Huanhuan Ma[1], Huabin Chi[1], Yizan Ma[1], Jing Yang[3], Sai Xie[1], Rui Zhang[1], Linying Liu[1], Xiaojun Su[1], Rongjie Lv[1], Aamir Hamid Khan[1], Jie Kong[3], Xiaoping Guo[2], Keith Lindsey[4], Ling Min[1*] and Xianlong Zhang[1]

Affiliations

[1] National Key Laboratory of Crop Genetic Improvement, Huazhong Agricultural University, Wuhan, Hubei 430070, China

[2] College of Plant Science and Technology, Huazhong Agricultural University, 430070 Wuhan, Hubei, China

[3] Institute of Economic Crops, Xinjiang Academy of Agricultural Sciences, Xinjiang 830091, China

[4] Department of Biosciences, Durham University, United Kingdom

* To whom correspondence should be addressed.
Tel: +86-2787283955, E-mails: lingmin@mail.hzau.edu.cn.

Key words: cotton; male sterility; exine formation; no spine pollen; polygalacturonase; de-esterified homogalacturonan

This file includes:

1. Supplementary Methods

2. Supplementary Figures S1 to S18

3. Supplementary Table S1

4. Supplementary References

Supplementary Methods

Tissue Collection and RNA Extraction

For real-time PCR and semiquantitative RT-PCR, seeds of *G. hirsutum* cv YZ1 were germinated on half-strength Murashige and Skoog medium (Min et al., 2013) supplemented with 1.5% (w/v) glucose and solidified with 0.25% (w/v) phytagel (Sigma, USA) at 28 °C in the dark for 3 d. Part of the seedlings were transferred to the nutrient solution, and the root and stems were collected after cultured for 10 d at 28±2 °C under a 14 h photoperiod. The other seedlings were transferred to soil and grown in the greenhouse for collecting the tissues of leaf, stage 1-13 anther, pistil, 0-day ovule and 5-day ovule. All collected samples were immediately flash-frozen in liquid nitrogen. Total RNA was extracted using a modified guanidine thiocyanate method (Zhu et al., 2005). 1 μg to 2 μg of total RNA was used for cDNA synthesis using Moloney Murine Leukemia Virus Reverse Transcriptase (M-MLV, Promega, USA). All primer sequences can be found in Supplemental Table S1.

GhNSP cloning and Bioinformatics Analysis

Full-lengths sequence of *GhNSP* were amplified with primer pairs *GhNSP*-full-S/AS by using the cultivar YZ1 cDNA as the PCR template, and cloned into the pDONR™/Zeo (invitrogen) by Gateway cloning technology. Full-lengths *GhNSP* cDNA without a stop codon were also isolated using the

primers *GhNSP*-full-S and *GhNSP*-full-wtaa-AS, and cloned into the pDONR™/Zeo. The primers used in this study are listed in Supplemental Table S1. To evaluate the potential functions of *GhNSP*, the protein sequence was functionally annotated using InterPro (http://www.ebi.ac.uk/interpro) (Mitchell et al., 2019).

Phylogenetic Analyses

The *GhNSP* homologous sequences of *Physcomitrella patens*, *Sphagnum fallax*, *Orzya sativa*, *Sorghum bicolor*, *Zea mays*, *Gossypium raimondii, Theobroma cacao, Populus trichocarpa, solanum lycopersicum, Vitis vinifera and Ricinus communis* were obtained from the Phytozome v12 database (https://phytozome.jgi.doe.gov/pz/portal.html) and aligned using the Clustalx software (http://www.clustal.org/). Phylogenetic trees for proteins with 1000 bootstrap replicates were constructed using the maximum likelihood method in MEGA7 (Kumar et al., 2016).

RNA-seq analysis

The raw data of RNA-seq derived from Wu *et al.* (2015), and the clean reads were mapped to the TM-1 genome (Wang et al., 2019) using HISAT2/2.1.0 (Kim et al., 2015). Then the gene expression level was calculated using FPKM (Fragments per Kilobase of exon model per Million mapped reads) with StringTie (Pertea et al., 2016). The heatmaps were drew using R (https://www.r-project.org/).

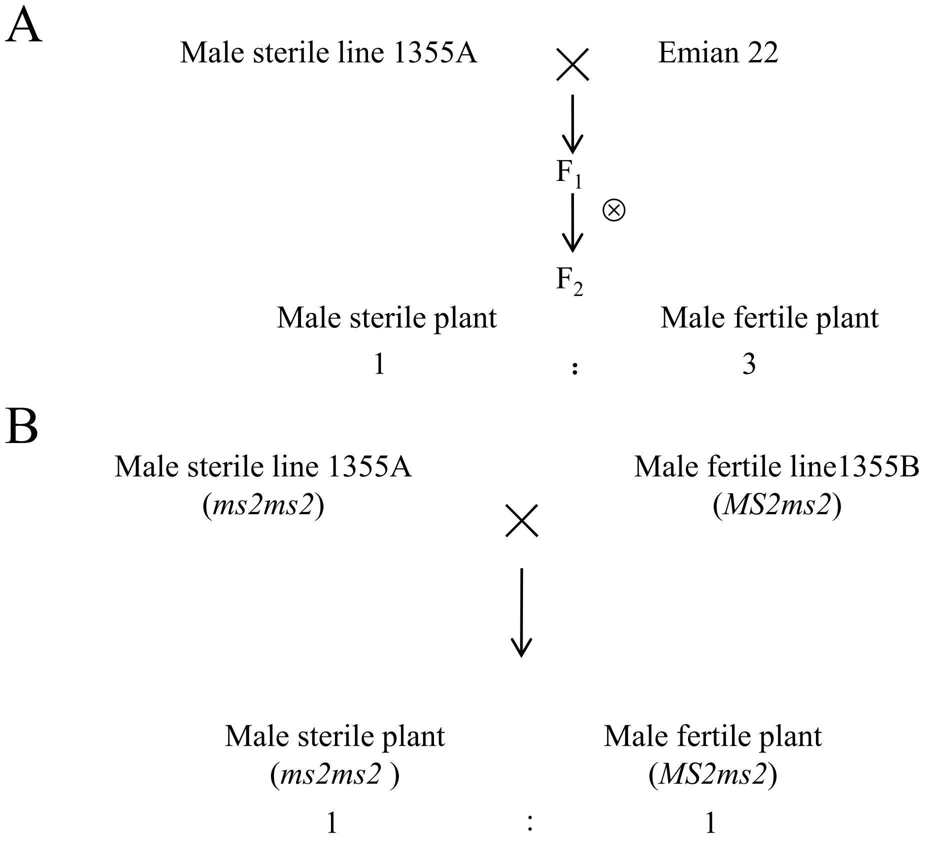

Supplementary Fig. S1 Populations developed for map-based cloning of the fertility restorer *GhNSP* for 1355A male sterile line.

(A) The F_2 population was constructed by using 1355A crossed with Emian 22.

(B) The segregation population was constructed by using 1355A crossed with 1355B.

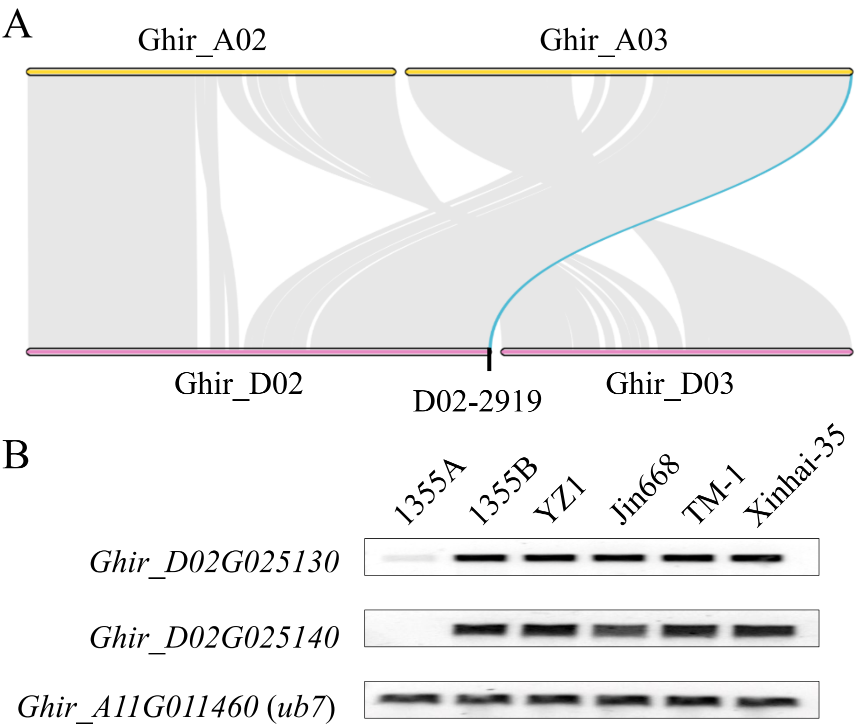

Supplementary Fig. S2 Synteny analysis and molecular test of the candidate region.

(A) Synteny analysis of the Chromosome Ghir_A02, Ghir_A03, Ghir_D02 and Ghir_D03, on the basis of the genome sequence of *G. hirsutum* cv TM-1 showing that there were syntenic blocks between the Ghir_D02 and Ghir_A03 in the deletion region. D02-2919, SSR (simple sequence repeat) marker.

(B) To prove that *Ghir_D02G025140* was deleted in 1355A plants, the molecular test using the gene-specific primers of *Ghir_D02G025130*, *Ghir_D02G025140* and *Ghir_A11G011460* (*ub7*) and the DNA derived from 1355A, 1355B, YZ1, Jin668, TM-1 and *G. barbadense* cv Xinhai-35 plants were performed, and showed that *Ghir_D02G025140* is deleted in 1355A plants.

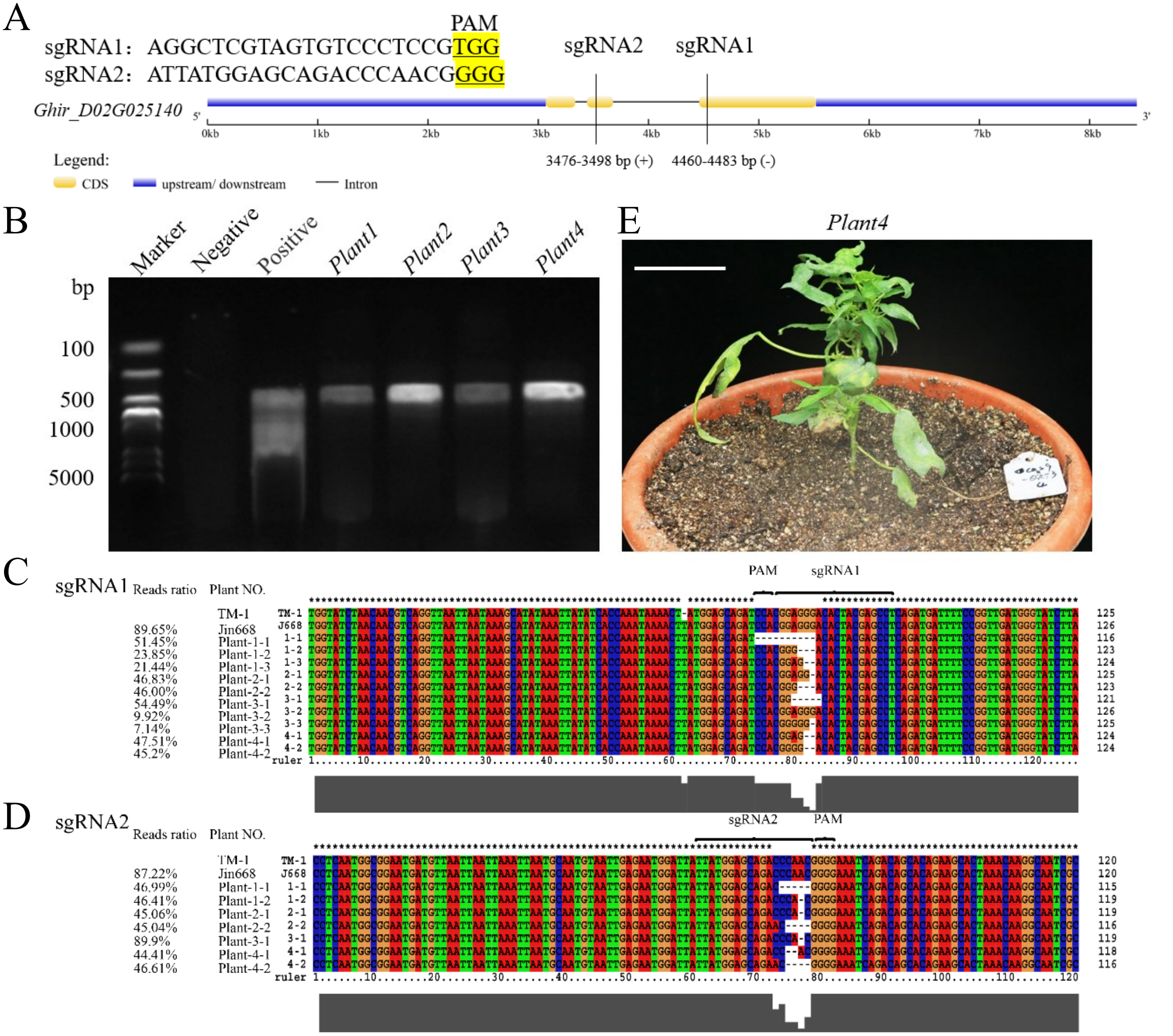

Supplementary Fig. S3 A schematic for generating the mutant of *GhNSP*.

(A) *Ghnsp* mutation was created by the CRISPR/Cas9 system, and the sgRNA1 and sgRNA2 are the target sites. The sequence and location of sgRNA1 and sgRNA2 are shown. The PAM is highlighted in yellow background and underlined. (+), Forward; (-), Reverse. PAM, protospacer adjacent motif.

(B) Transgenic plants (T_0) were detected by using the primer of u6-7s and inf Gh*NSP*-CRISPR-AS in the pRGEB32-GhU6.7-NPT II vector, it indicated that all plants are positive for the presence of the transgene.

(C) and (D) Use of the Hi-TOM platform to track mutations induced by CRISPR/Cas9 systems. Genome editing at sgRNA1 (C) and sgRNA2 (D) in transgenic plants include *plant1*, *plant2*, *plant3* and *plant4*. The sgRNA and PAM is highlighted.

(E) *plant4* showed abnormal development. Bars, 5 cm.

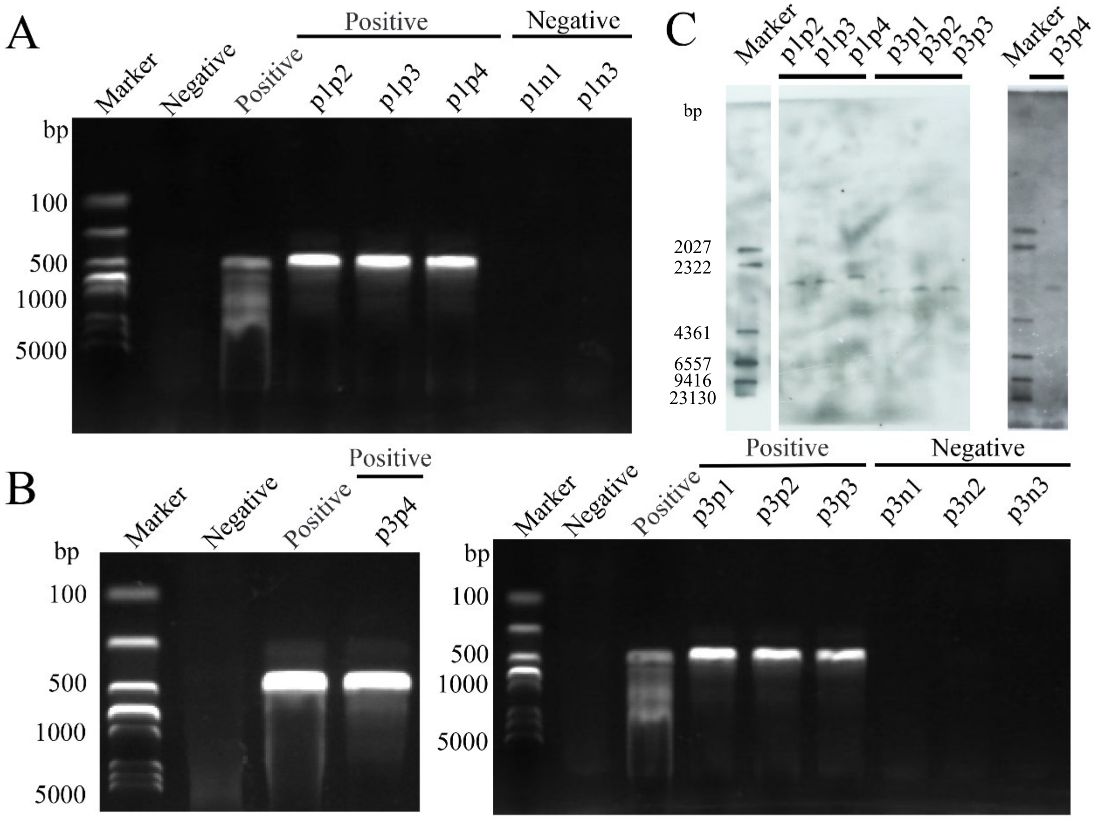

Supplementary Fig. S4 PCR and Southern blotting analysis in the individuals of the F_1 generation of *Ghnsp* mutants crossed with WT (Jin668).

(A) The individuals of F_1 generation of *plant1* crossed with WT were detected using the primers of u6-7s and inf *GhNSP*-CRISPR-AS in the pRGEB32-GhU6.7-NPT II vector, and showed that there are three positive plants and two negative plants. p1p2-p1p4, the individuals from positive plants; p1n1 and p1n3, the individuals from negative plants.

(B) The individuals of F_1 generation of *plant3* crossed with WT were detected by using the prime of u6-7s and inf *GhNSP*-CRISPR-AS in the pRGEB32-GhU6.7-NPT II vector, and showed that there are four positive plants and three negative plants. p3p1-p3p4, the individuals from positive plants; p3n1-p3n3, the individuals from negative plants.

(C) Southern blotting analysis of the positive plants of the F_1 generation were performed, and showed that single copy of T-DNA inserted in *plant1* and *plant3*. The probe was NPT II.

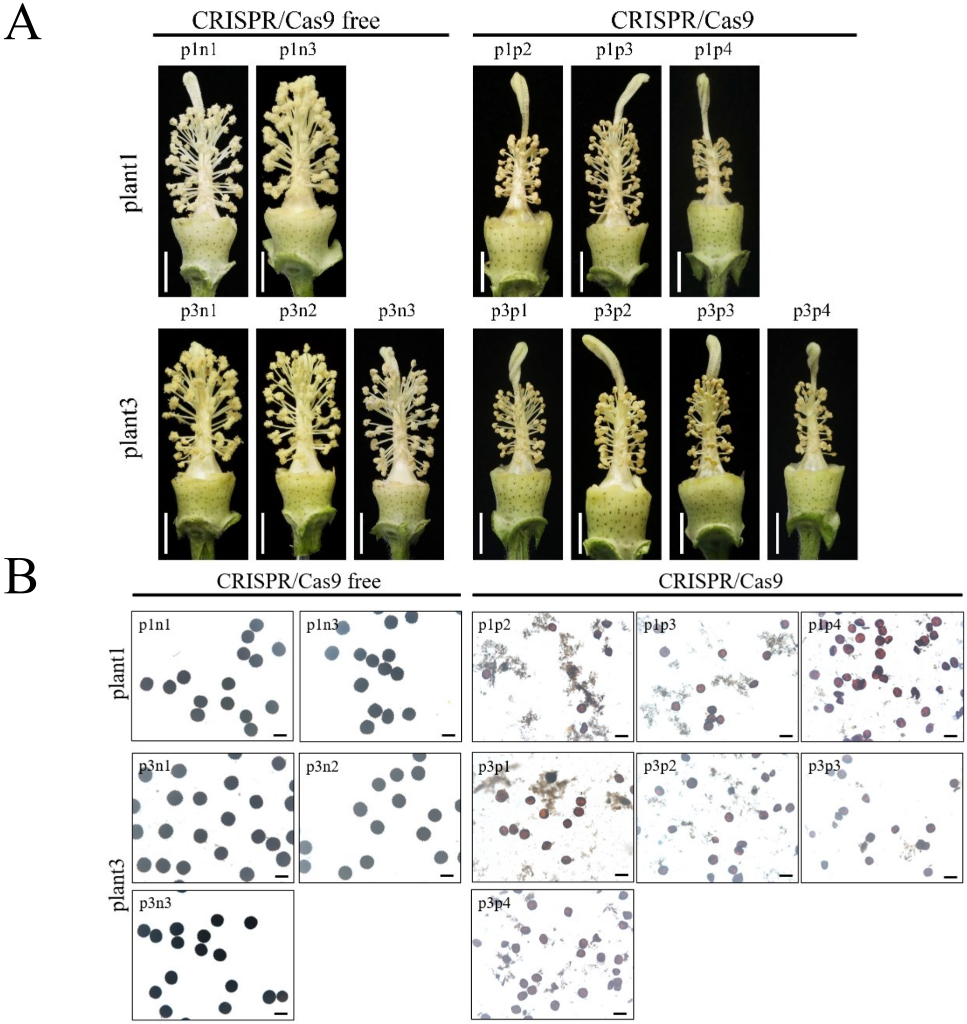

Supplementary Fig. S5 Phenotypic comparison between the individual of the F_1 generation of the transgenic plants (T_0) crossed with Jin668.

(A) Anthers of the individual of the F_1 generation of the transgenic plants (T_0) crossed to Jin668 were shown. Anthers of all CRISPR/Cas9 free plants were dehiscent, while anthers of CRISPR/Cas9 plants were indehiscent.

(B) Pollen grains of the individual of the F_1 generation of the transgenic plants (T_0) crossed with Jin668 stained with 1% I_2-KI solution, showing that all CRISPR/Cas9 free plants were male fertile (black pollen), and all CRISPR/Cas9 containing plants were male sterile (red pollen). Bars, 1 cm in (A); 100 μm in (B).

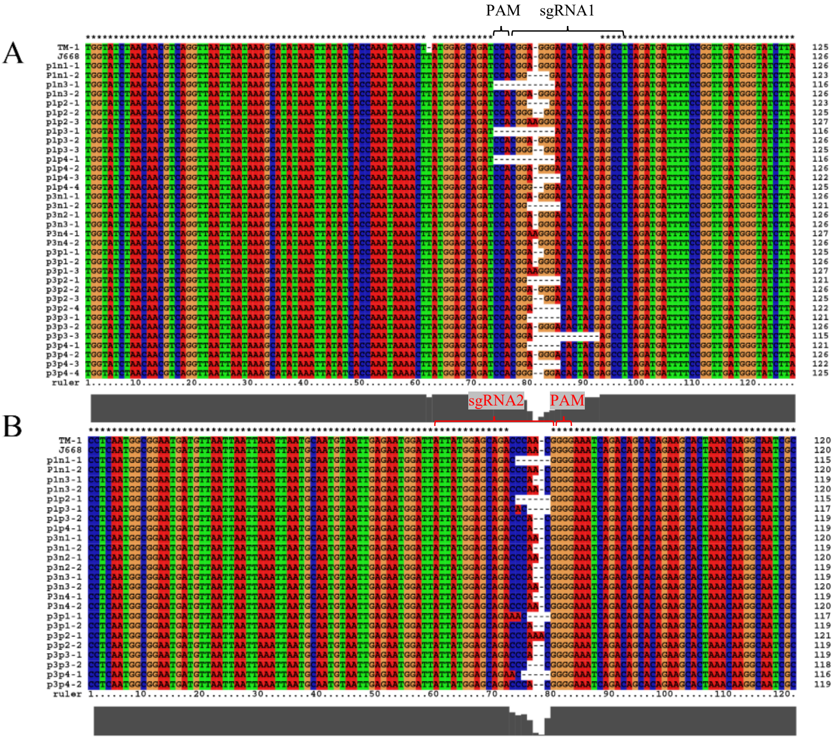

C

Plant ID	Genotype	
	sgRNA 1	sgRNA 2
Jin668	AA	BB
p1n1	Aa	Bb
p1n3	Aa	Bb
p1p2	aa	bb
p1p3	Aa	bb
p1p4	Aa	bb
p3n1	Aa	Bb
p3n2	AA	Bb
p3n3	AA	Bb
p3p1	Aa	bb
P3p2	Aa	bb
p3p3	Aa	bb
p3p4	Aa	bb

Supplementary Fig. S6 Genotypic analysis of the individual of the F_1 generation of the transgenic plants (T_0) crossed with Jin668.

(A) and (B) Use of Hi-TOM platform to track genome editing at sgRNA1 (A) and sgRNA2 (B) in the individual of the F_1 generation of the transgenic plants (T_0) crossed with Jin668. The sequence and location of sgRNA1 and sgRNA2 are shown. The sgRNA and PAM are highlighted. PAM, protospacer adjacent motif.

(C) Hi-TOM sequence results are summarized in the table. A\a represent sgRNA1 site; B\b represent sgRNA2 site;

AA and BB stand for wild-type in *GhNSP* locus; Aa and Bb stand for heterozygous genotype in *GhNSP* locus; aa and bb stand for mutant genotype in *GhNSP* locus.

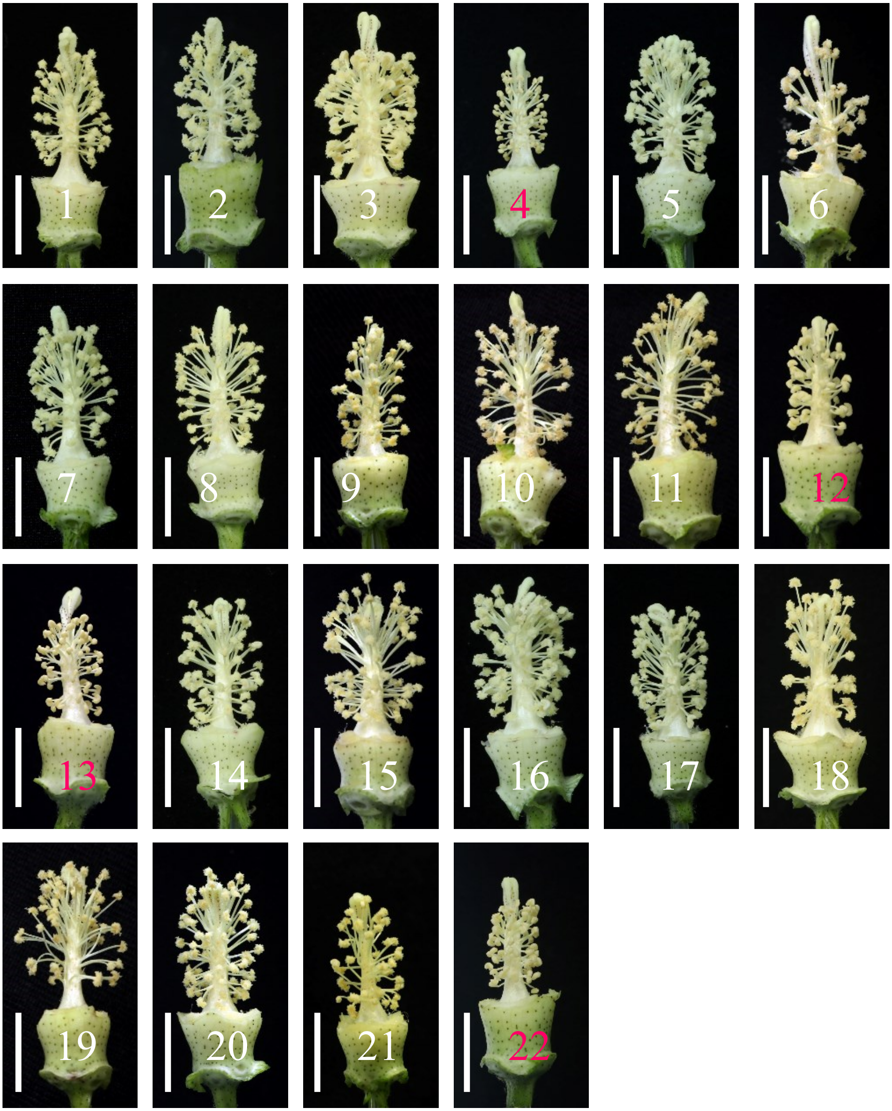

Supplementary Fig. S7 Phenotypic analysis of the individuals of S_1 population derived from p3n3 plant.

Phenotype analysis of the anthers from 22 individual of S_1 population derived from p3n3 plant revealed four individuals with indehiscent anthers. The number with white and bright red represent male fertile plant and male sterile plant, respectively. p3n3, the CRISPR/Cas9 free plants derived from the F_1 generation of the *plant3* mutant (T_0) crossed with WT. S_1 populations, the first progeny of p1n1 or p3n3 plant through self-pollination. Bars, 1 cm.

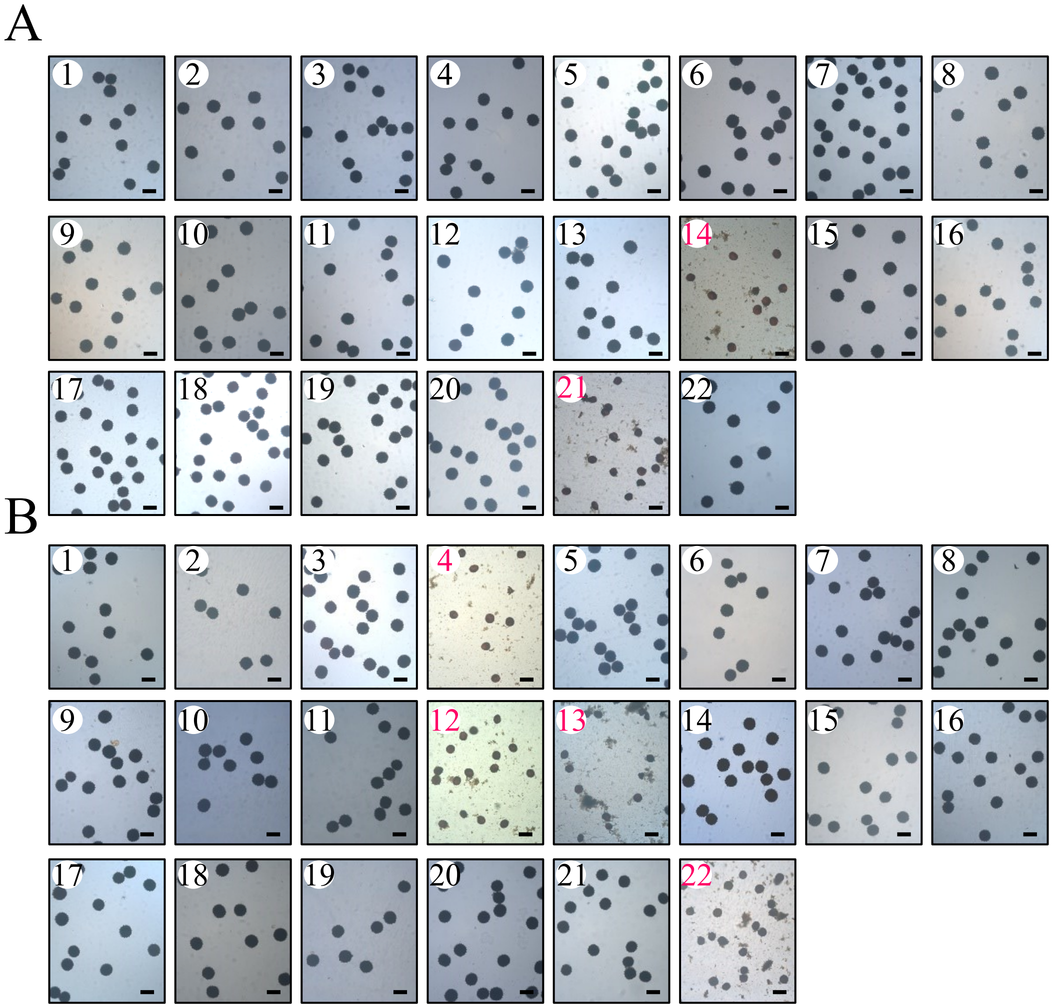

Supplementary Fig. S8 Pollen phenotypic analysis of the individuals of the S_1 populations derived from p1n1 plant (22 individuals) and p3n3 plant (22 individuals). (A) and (B) Pollen grains of the individuals of the S_1 populations derived from p1n1 plant (A) and p3n3 plant (B), stained with 1% I_2-KI. The male fertile pollen grains are dyed black, while the male sterile pollen grains are dyed red. There are two and four individuals were male sterility in the S_1 populations derived from p1n1 and p3n3, respectively. Which is consistent with the result of anther dehiscence. The number with black and bright red represent male fertile plant and male sterile plant, respectively. p1n1 and p3n3, the CRISPR/Cas9 free plants derived from the F_1 generation of the *plant1* and *plant3* mutant (T_0) crossed with WT, respectively. S_1 populations, the first progeny of p1n1 or p3n3 plant through self-pollination. Bars, 100 μm in (A) and (B).

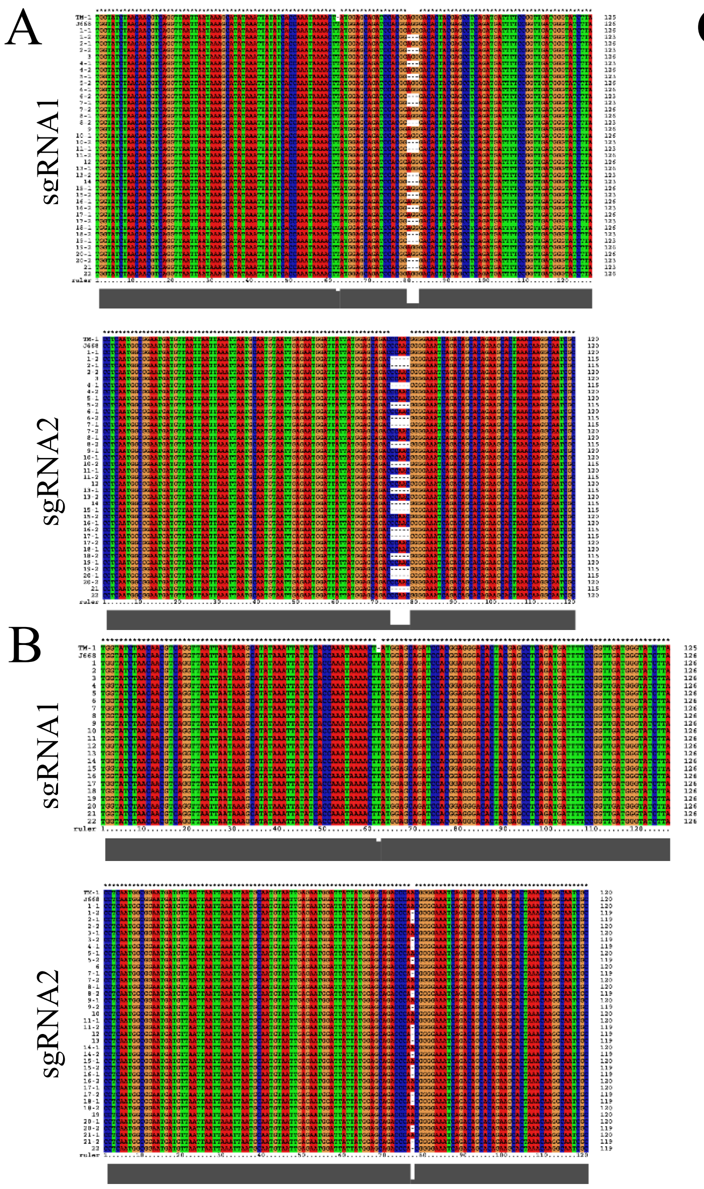

C

Plant ID	Genotype	
	sgRNA1	sgRNA2
Jin668	AA	BB
1	AA	Bb
2	AA	Bb
3	AA	Bb
4	AA	bb
5	AA	Bb
6	AA	BB
7	AA	Bb
8	AA	Bb
9	AA	Bb
10	AA	BB
11	AA	Bb
12	AA	bb
13	AA	bb
14	AA	Bb
15	AA	Bb
16	AA	Bb
17	AA	Bb
18	AA	Bb
19	AA	BB
20	AA	Bb
21	AA	Bb
22	AA	bb

Supplementary Fig. S9 Genotypic analysis of the individuals of the S_1 populations derived from p1n1 and p3n3.

(A) and (B) Use of Hi-TOM platform to track mutations induced by CRISPR/Cas9 systems. The Hi-TOM sequence results of the individuals of the S_1 populations derived from p3n3. (C) Genotypes summarized in the table. S_1 populations, the first progeny of p1n1 or p3n3 plant through self-pollination. A\a represent sgRNA1 site; B\b represent sgRNA2 site; AA and BB stand for wild-type in *GhNSP* locus; Aa and Bb stand for heterozygous genotype in *GhNSP* locus; aa and bb stand for homozygous mutant genotype in *GhNSP* locus. The orange characters represent the genotype of male sterile plant.

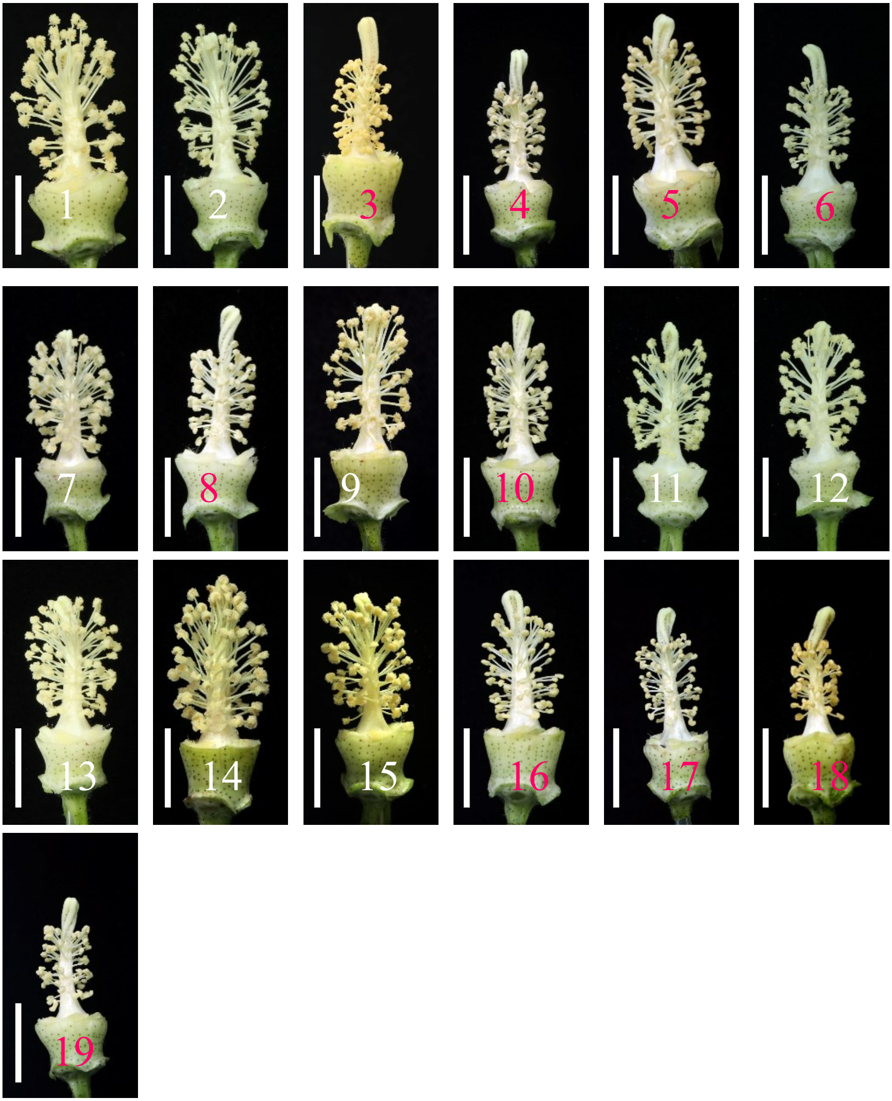

Supplementary Fig. S10 Phenotypic analysis of the individuals of C_1 population derived from p3n3.

Phenotype analysis of the anthers from 19 individuals of C_1 population derived from p3n3 plant revealed that 10 individuals with indehiscent anthers, and nine individuals with dehiscent anthers. p3n3, the CRISPR/Cas9 free plants derived from the F_1 generation of the *plant3* mutant (T_0) crossed with WT. C_1 populations, the first progeny of p1n1 or p3n3 plant crossed with 1355A plant. Bars, 1 cm.

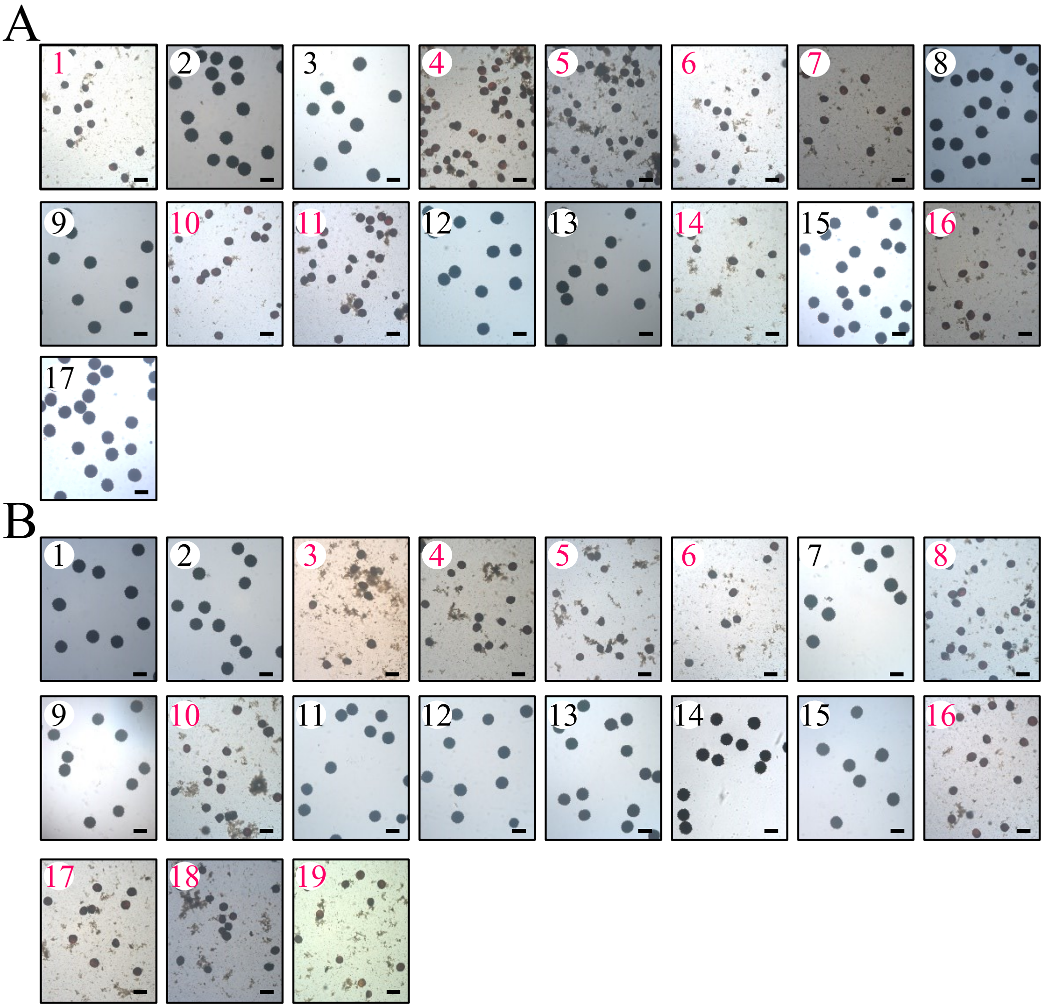

Supplementary Fig. S11 Pollen phenotypic analysis of the individuals of C_1 populations derived from p1n1 and p3n3. (A) and (B) Pollen grains of the individuals of C_1 populations derived from p1n1(A) and p3n3 (B), stained with 1% I_2-KI. The male fertile pollen grains are dyed black, while the male sterile pollen grains are dyed red. The number with black and bright red represent male fertile plant and male sterile plant, respectively. p1n1 and p3n3, the CRISPR/Cas9 free plants derived from the F_1 generation of the *plant 1* and *plant3* mutant (T_0) crossed with WT, respectively. C_1 populations, the first progeny of p1n1 or p3n3 plant crossed with 1355A plant. Bars, 100 μm in (A) and (B).

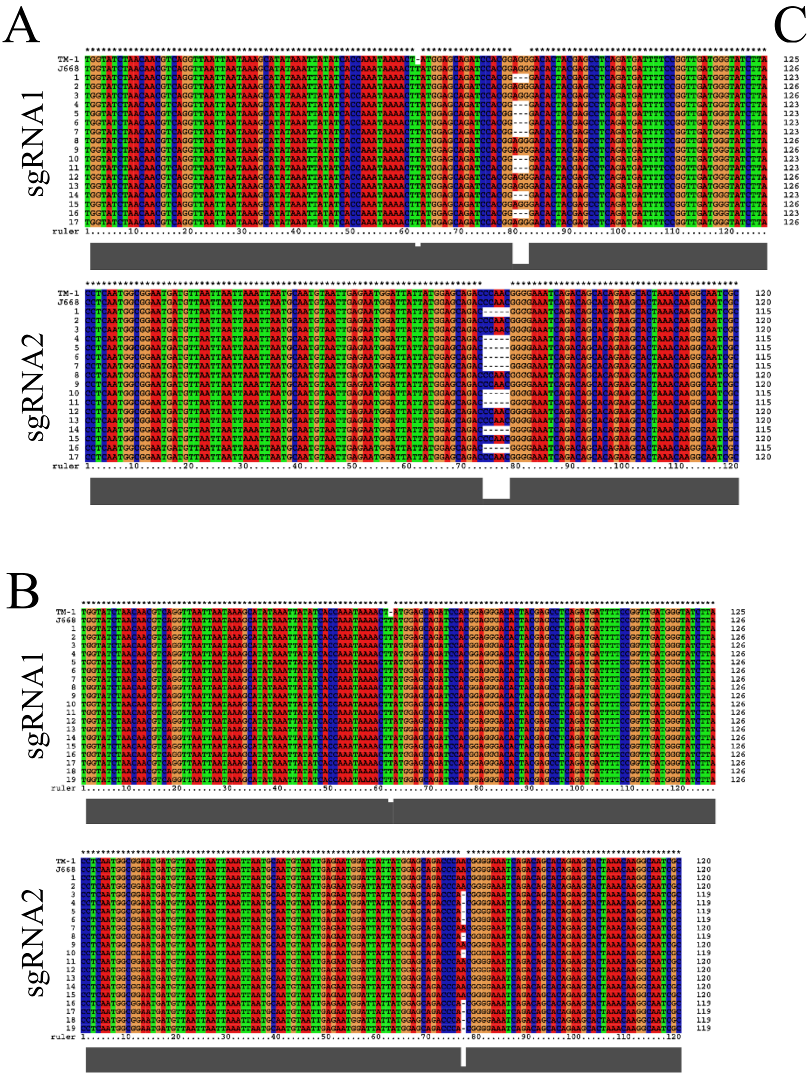

C

Plant ID	Genotype	
	sgRNA1	sgRNA2
Jin668	AA	BB
1	AA	BB
2	AA	BB
3	AA	bb
4	AA	bb
5	AA	bb
6	AA	bb
7	AA	BB
8	AA	bb
9	AA	BB
10	AA	bb
11	AA	BB
12	AA	BB
13	AA	BB
14	AA	BB
15	AA	BB
16	AA	bb
17	AA	bb
18	AA	bb
19	AA	bb

Supplementary Fig. S12 Genotypic analysis of the individuals of C_1 population derived from p1n1 and p3n3.

(A) and (B) Use of Hi-TOM platform to track mutations created by CRISPR/Cas9 systems. The genome editing at sgRNA1 and sgRNA2 in the individuals of C_1 populations derived from p1n1 (A) and p3n3 (B) crossed to 1355A. And the Hi-TOM sequence results of the individuals of first-generation of p3n3 (C) are summarized in the table. A\a represent sgRNA1 site; B\b represent sgRNA2 site; AA and BB stand for wild-type in *GhNSP* locus; Aa and Bb stand for heterozygous genotype in *GhNSP* locus; aa and bb stand for homozygous mutant genotype in *GhNSP* locus. The orange characters represent the genotype of male sterile plant. p1n1 and p3n3, the CRISPR/Cas9 free plants derived from the *plant1* and *plant3* mutant crossed with WT, respectively. C_1 populations, the first progeny of p1n1 or p3n3 plant crossed with 1355A plant.

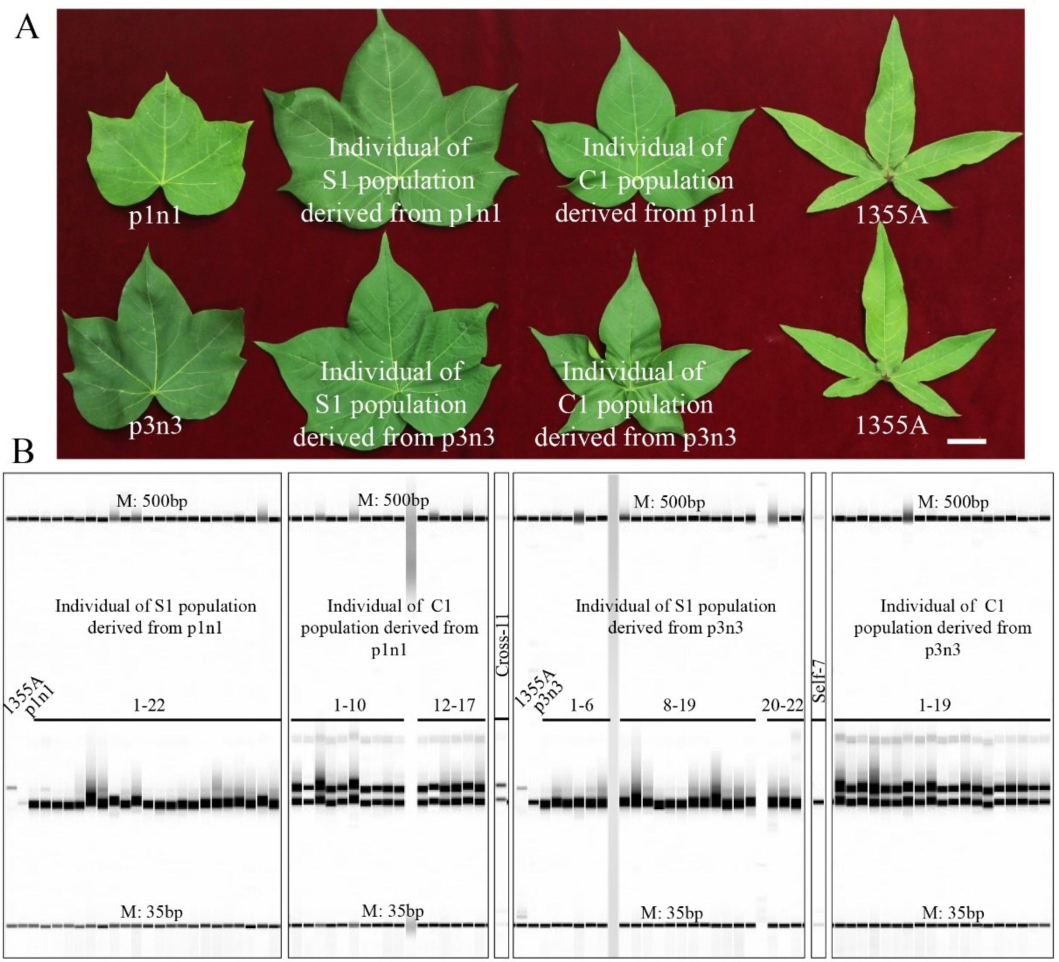

Supplementary Fig. S13 Molecular test for the population for co-segregation test (S_1 populations) and allele prediction (C_1 populations).

(A) Leaf shape is different between mutant and 1355A plants. The *Ghnsp* mutant plants and the plants of S_1 populations show normal leaves, the 1355A plants show okra leaf, and the individual of the C_1 populations show subokra. S_1 populations, the first progeny of p1n1 or p3n3 plant through self-pollination. C_1 populations, the first progeny of p1n1 or p3n3 plant crossed with 1355A plant. Bar = 2 cm.

(B) Molecular test of the population for the co-segregation test and allele prediction using the SSR marker SWU07345. Red arrow shows the polymorphism of SWU07345 marker in the parents. M, marker; bp, base pair.

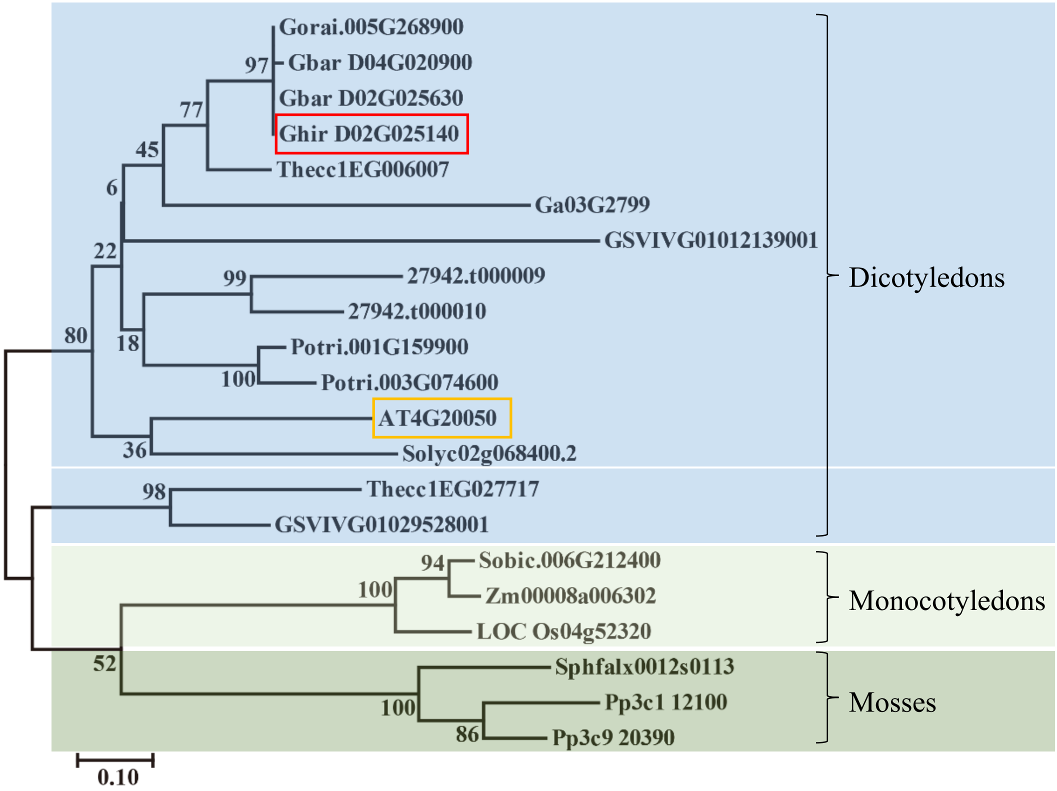

Supplementary Fig. S14 Phylogenetic tree of GhNSP (Ghir_D02G025140, red box) and its homologs from other species.

A maximum likelihood phylogenetic tree was constructed using MEGA 7 to determine the evolutionary relationships among GhNSP and its homologues. A total of 20 closely related sequences were retrieved from moss [*Physcomitrella patens* (Pp3c920390 and Pp3c112100) and *Sphagnum fallax* (Sphfalx0012s0113)], monocotyledonary [*Orzya sativa* (LOC Os04g52320), *Sorghum bicolor* (Sobic.006G212400) and *Zea mays* (Zm00008a006302)], and dicotyledonary species [*Gossypium barbadense* (GbarD02G025630 and GbarD04G020900), *Gossypium arboretum* (Ga03G2799), *Gossypium raimondii* (Gorai.005G268900), *Theobroma cacao* (Thecc1EG006007 and Thecc1EG027717), *Arabidopsis* (AT4G20050, QRT3, orange box), *Populus trichocarpa* (Potri.001G159900 and Potri.003G074600), *solanum lycopersicum* (Solyc02g068400.2), *Vitis vinifera* (GSVIVG01012139001 and GSVIVG01029528001) and *Ricinus communis* (27942.t000009 and 27942.t000010)].

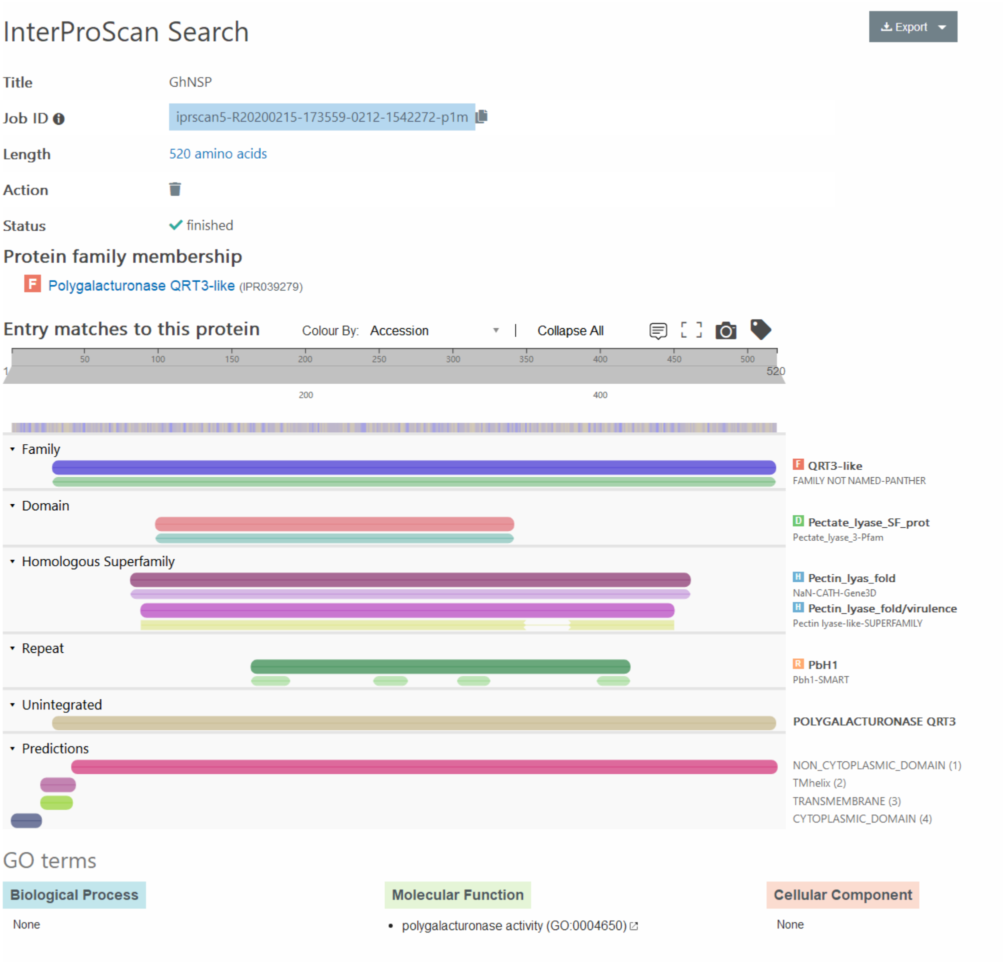

Supplementary Fig. S15 Using InterProScan to search conserved domains of the GhNSP protein.

The GhNSP protein sequence was functionally annotated using InterPro, showing that GhNSP contains consensus pectate_lyase_3 domain.

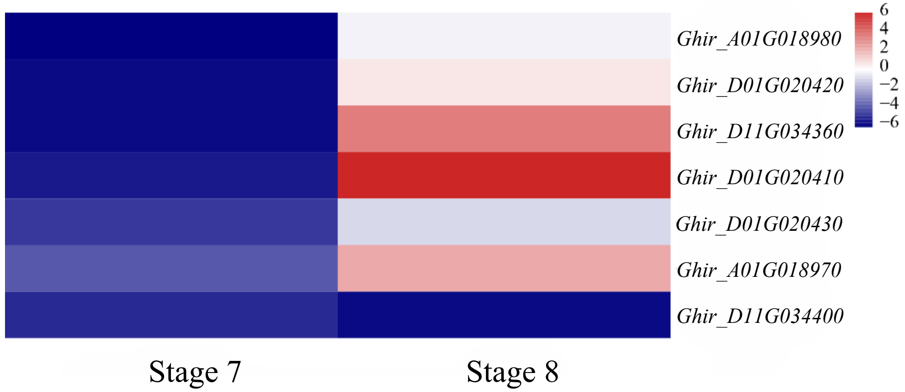

Supplementary Fig. S16 The expression pattern analysis of the *AGP23* at 1355AB lines during the stage 7 and stage 8.

The *APG23* homologous genes in *G. hirsutum* were down-regulated at stage 7, and slightly up-regulated at stage 8 in 1355A male sterile lines.

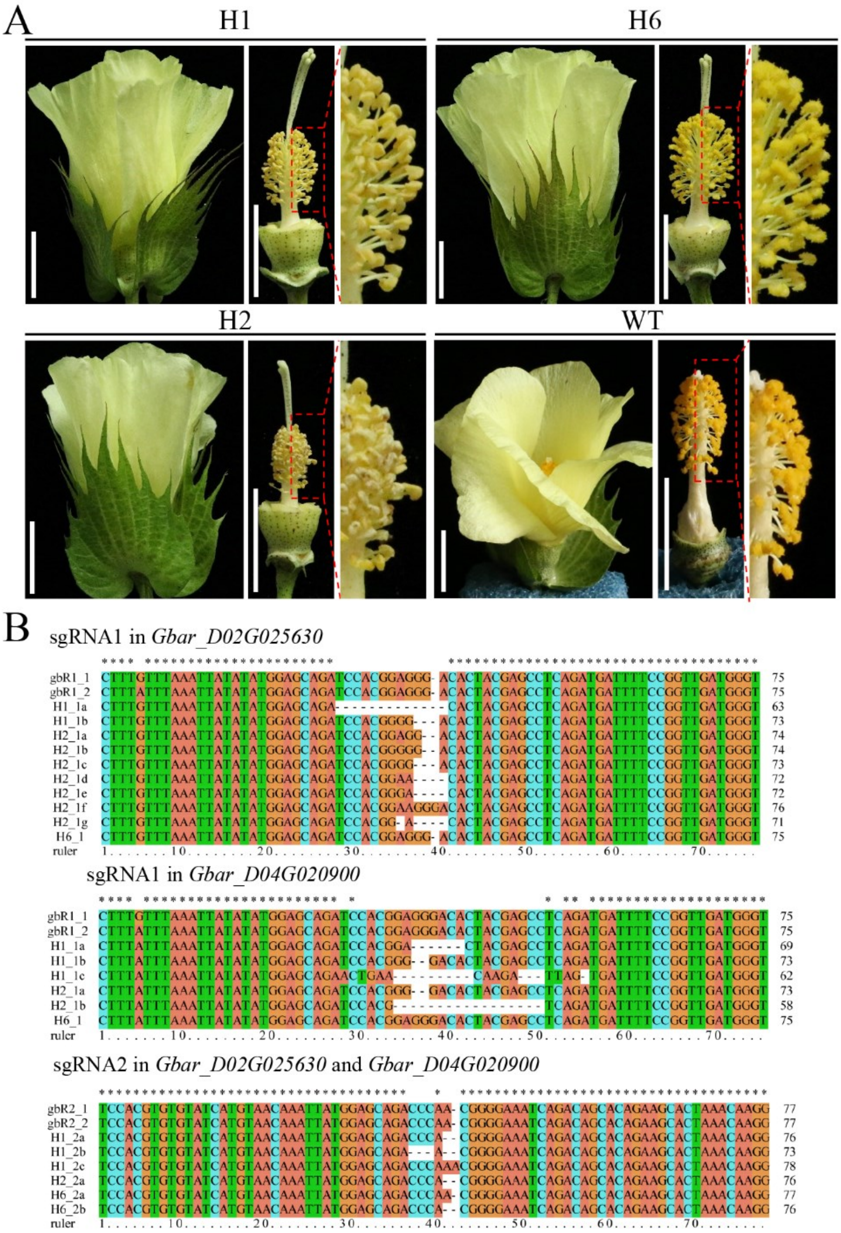

C

Plant ID	*Gbar_D02G025630*		*Gbar_D04G020900*	
	sgRNA1	sgRNA2	sgRNA1	sgRNA2
H1	aa	bb	Aa	bb
H2	aa	bb	aa	bb
H6	AA	Bb	AA	Bb

Supplementary Fig. S17 The strategy for breeding various male sterile lines in *Gossypium barbadense*.

(A) Phenotypic comparison between the *G. barbadense* (WT) and the mutations induced by the CRISPR/Cas9 system (*G. hirsutum* T_0 mutant crossed with *G. barbadense* to produce F_1 plants). The flowers of plants WT and mutants were shown, with petals removed. The anthers of H1 and H2 were indehiscence, and the anthers of H6 were dehiscence, which suggest that the H1 and H2 plants are male sterile, and the H6 plant is male fertile. Bars, 2 cm.

(B) Use of Hi-TOM platform to track mutations induced by CRISPR/Cas9 systems.

(C) Genome editing at sgRNA1 and sgRNA2 of *Gbar_D02G025630* and *Gbar_D04G020900* in the individuals of F_1 generation of *G. barbadense* crossed with the mutant *plant3* (*G. hirsutum* cv Jin668). H1, H2 and H6: the progeny of the mutant *plant3* (as maternal parent) crossed with *G. barbadense* cv xinhai-35. A\a represent sgRNA1 site; B\b represent sgRNA2 site; AA and BB stand for wild-type in target site; Aa and Bb stands for heterozygous genotype at the target site; aa and bb stand for homozygous at the target site.

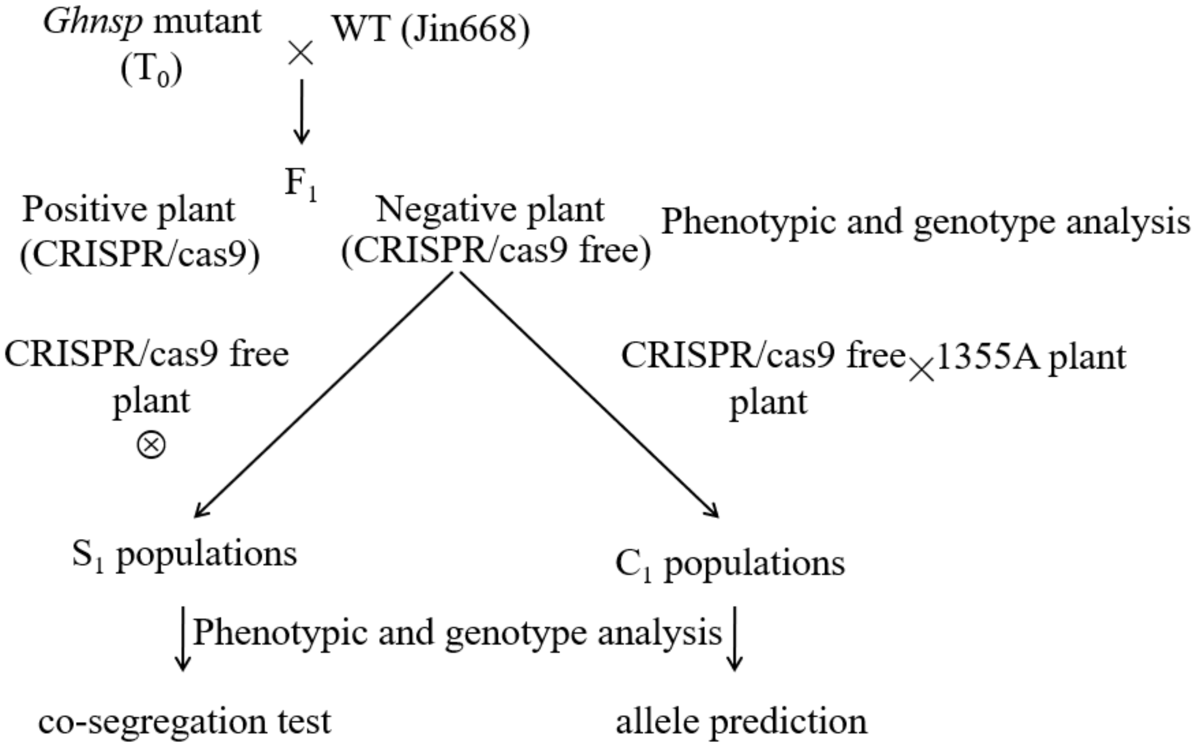

Supplementary Fig. S18 Population developed for co-segregation test and allele prediction.

The *Ghnsp* mutant (T_0) was crossed with WT, and generated F_1 populations. The CRISPR/cas9 free plants were selected in F_1 populations. The co-segregation populations (S_1 populations) were generated through self-pollination by using the CRISPR/Cas9 free plants, and the allele prediction populations (C_1 populations) were generated using the CRISPR/Cas9 free plants crossed with 1355A plants.

Supplementary Table S1. Oligonucleotides used in this study

Primer name	primer sequence (5'-3')	Destination
SSR2-7-S	GAGGTTTAGGTTTACTGCTTGT	**SSR marker for mapping**
SSR2-7-AS	ACATTTAGTGGAGGGGTTTTA	**SSR marker for mapping**
D02_2901-S	ATGACATTGGGTGGCTGCTT	**SSR marker for mapping**
D02_2901-AS	GTTCAATGGCGTTACAGGCG	**SSR marker for mapping**
D02_2910-S	TTTTCAACCGTTTCATTGTAGCT	**SSR marker for mapping**
D02_2910-AS	ACAAGCAGTATCTCGTTCCAG	**SSR marker for mapping**
D02_2919-S	TTGGCTGATCGAACGGTCTG	**SSR marker for mapping**
D02_2919-AS	AGGACACACAGATGCGATCG	**SSR marker for mapping**
D02_2922-S	TGGGTGGGGGTTTTGTTCAT	**SSR marker for mapping**
D02_2922-AS	TGCTACTACTACTTGCTGCAGG	**SSR marker for mapping**
GhNSP-CRISPR-1-AS	CGGAGGGACACTACGAGCCTtgcaccagccgggaat	**CRISPR/Cas9 vector**
GhNSP-CRISPR-2-S	AGGCTCGTAGTGTCCCTCCGgttttagagctagaaata	**CRISPR/Cas9 vector**
GhNSP-CRISPR-2-AS	CGTTGGGTCTGCTCCATAATtgcaccagccgggaat	**CRISPR/Cas9 vector**
inf GhNSP-CRISPR-AS	ttctagctctaaaacCGTTGGGTCTGCTCCATAAT	**CRISPR/Cas9 vector**
u6-7s	TGTGCCACTCCAAAGACATCAG	**CRISPR/Cas9 vector**
gRNA1-Hi-Tom-S	GGAGTGAGTACGGTGTGCTGGTATCTAACAACGTCAGGTTAAT	**primer for Hi-TOM for *G. hirsutum***
gRNA1-Hi-Tom-AS	GAGTTGGATGCTGGATGGTAAGATACCCATCAACCGGAAA	**primer for Hi-TOM for *G. hirsutum***
gRNA2-Hi-Tom-S	GGAGTGAGTACGGTGTGCCCTCAATGGCGGAATGATGTT	**primer for Hi-TOM for *G. hirsutum***
gRNA2-Hi-Tom-AS	GAGTTGGATGCTGGATGGGCGATTGCCTTGTTTAGTGCTT	**primer for Hi-TOM for *G. hirsutum***
gRNA1-Hi-Tom-gb-S	GGAGTGAGTACGGTGTGCATTTGCTGAGTTGGGTATTT	**primer for Hi-TOM for G. barbadense**
gRNA1-Hi-Tom-gb-AS	same to gRNA1-Hi-Tom-AS	**primer for Hi-TOM for G. barbadense**
gRNA2-Hi-Tom-gb-S	GGAGTGAGTACGGTGTGCTGTAATTGAGAATGGATTTG	**primer for Hi-TOM for G. barbadense**
gRNA2-Hi-Tom-gb-AS	same to gRNA2-Hi-Tom-AS	**primer for Hi-TOM for G. barbadense**
GhNSP-S	GGGTCTGTTGAAGGGAATAGCA	**RT-PCR, qRT-PCR and *in situ* hybridization**
GhNSP-AS	TGGAGTAAATGGGTGAGGGAAA	**RT-PCR, qRT-PCR and *in situ* hybridization**
SWU07345_F	AACGGTGTTGGGACTGAGTT	**SSR marker for Leaf shape**
SWU07345_R	AGGTTTCGGTTGGAGAAGAA	**SSR marker for Leaf shape**

GhNSP-full-S	GGGGACAAGTTTGTACAAAAAAGCAGGCTCAATGTCTCTCTCTCAAAACGGAGA	**cloning *GhNSP***
GhNSP-full-AS	GGGGACCACTTTGTACAAGAAAGCTGGGTCTTAAGCTTGATCCACCGTGAC	**cloning *GhNSP***
GhNSP-full-wtaa-AS	GGGGACCACTTTGTACAAGAAAGCTGGGTCAGCTTGATCCACCGTGAC	**cloning *GhNSP***

Supplementary References

Kim, D., Langmead, B., and Salzberg, S.L. (2015). HISAT: a fast spliced aligner with low memory requirements. *Nat. Methods* **12,** 357-360.

Kumar, S., Stecher, G., and Tamura, K. (2016). MEGA7: Molecular Evolutionary Genetics Analysis Version 7.0 for Bigger Datasets. *Mol. Biol. Evol.* **33,** 1870.

Min, L., Zhu, L., Tu, L., Deng, F., Yuan, D., and Zhang, X. (2013). Cotton GhCKI disrupts normal male reproduction by delaying tapetum programmed cell death via inactivating starch synthase. *Plant J.* **75,** 823-835.

Mitchell, A.L., Attwood, T.K., Babbitt, P.C., Blum, M., Bork, P., Bridge, A., Brown, S.D., Chang, H.-Y., El-Gebali, S., Fraser, M.I., Gough, J., Haft, D.R., Huang, H., Letunic, I., Lopez, R., Luciani, A., Madeira, F., Marchler-Bauer, A., Mi, H., Natale, D.A., Necci, M., Nuka, G., Orengo, C., Pandurangan, A.P., Paysan-Lafosse, T., Pesseat, S., Potter, S.C., Qureshi, M.A., Rawlings, N.D., Redaschi, N., Richardson, L.J., Rivoire, C., Salazar, G.A., Sangrador-Vegas, A., Sigrist, C.J.A., Sillitoe, I., Sutton, G.G., Thanki, N., Thomas, P.D., Tosatto, S.C.E., Yong, S.-Y., and Finn, R.D. (2019). InterPro in 2019: improving coverage, classification and access to protein sequence annotations. *Nucleic Acids Res.* **47,** D351-D360.

Pertea, M., Kim, D., Pertea, G.M., Leek, J.T., and Salzberg, S.L. (2016). Transcript-level expression analysis of RNA-seq experiments with HISAT, StringTie and Ballgown. *Nat. Protoc.* **11,** 1650-1667.

Wang, M., Tu, L., Yuan, D., Zhu, D., Shen, C., Li, J., Liu, F., Pei, L., Wang, P., Zhao, G., Ye, Z., Huang, H., Yan, F., Ma, Y., Zhang, L., Liu, M., You, J., Yang, Y., Liu, Z., Huang, F., Li, B., Qiu, P., Zhang, Q., Zhu, L., Jin, S., Yang, X., Min, L., Li, G., Chen, L.-L., Zheng, H., Lindsey, K., Lin, Z., Udall, J.A., and Zhang, X. (2019). Reference genome sequences of two cultivated allotetraploid cottons, Gossypium hirsutum and Gossypium barbadense. *Nat. Genet.* **51,** 224-229.

Wu, Y., Min, L., Wu, Z., Yang, L., Zhu, L., Yang, X., Yuan, D., Guo, X., and Zhang, X. (2015). Defective pollen wall contributes to male sterility in the male sterile line 1355A of cotton. *Sci. Rep.* **5,** 9608.

Zhu, L.F., Li-Li, T.U., Zeng, F.C., Liu, D.Q., and Zhang, X.L. (2005). An Improved Simple Protocol for Isolation of High Quality RNA from Gossypium spp. Suitable for cDNA Library Construction. *Acta Agronomica Sinica* **31,** 1657-1659.
